# Evidence-Calibrated Reanalysis of an Otosclerosis Locus Generates a Testable Myeloid State Hypothesis

**DOI:** 10.3390/genes17101277

**Published:** 2026-10-14

**Authors:** Weijun Ma, Ying Cheng, Heng Li, Hui Lv

**Affiliations:** 1 Department of Otolaryngology-Head and Neck Surgery, The Second Affiliated Hospital of Xi’an Jiaotong University, Xi’an 710004, China; smart81110@mail.xjtu.edu.cn (Y.C.); 18829075058@163.com (H.L.); 18165325105@163.com (H.L.); 2 Shaanxi Provincial Key Laboratory for Precision Diagnosis and Treatment of Otorhinolaryngology, Xi’an 710004, China

**Keywords:** otosclerosis, osteoclast, colocalization, chromatin conformation, single-cell RNA sequencing, multi-omics

## Abstract

**Background**: Otosclerosis is a focal disorder of otic capsule bone remodeling, but variant-to-cell-state mechanisms remain unresolved. **Methods**: We performed an entirely secondary hypothesis-generating analysis of otosclerosis GWAS, osteoclast cis-eQTL, epigenomic, pooled Micro-C, and human single-cell RNA-sequencing datasets. **Results**: At a data-rich chromosome 13 locus selected for cross-layer tractability rather than prospective genome-wide superiority, colocalization was compatible with a shared regional signal under the conventional prior but was strongly prior-sensitive and did not resolve *COL4A2-AS2*, *COL4A2*, or *RAB20* as a unique effector transcript. Seven credible variants overlapped one mature osteoclast ATAC element, and merged osteoclast loop calls connected the risk/*COL4A2-AS2* interval with *RAB20*; related PBMC contacts and the absence of donor-resolved matrices precluded an osteoclast-specific regulatory claim. In a separate 12-donor bone metastasis dataset lacking chr13 genotypes, *RAB20* marked an AP1/*CTNNB1*-associated myeloid state, while a four-donor non-tumor femoral head dataset provided only directional cross-context prioritization of a *CXCL8*-*CXCR2* candidate axis. A simplified comparison across all 27 reported GWAS loci showed that the chr13 locus was among loci with credible variants in mature osteoclast open chromatin but was not uniquely strongest across the examined annotations. **Conclusions**: No analyzed dataset jointly measured the risk haplotype and the *RAB20*-associated state, and no raw dataset sampled an otosclerosis lesion. Thus, the genetic/topological and transcriptomic observations remain separate evidence blocks that generate a falsifiable myeloid state hypothesis rather than establish a mechanistic or causal pathway.

## 1. Introduction

Otosclerosis is characterized by focal dysregulated bone turnover in the otic capsule, most commonly near the anterior margin of the oval window, with subsequent fixation of the stapes and conductive hearing loss. Histologically active lesions may contain vascular, cellular, and fibro-osseous tissue, whereas later lesions become sclerotic [1,2,3,4]. These features distinguish otosclerosis from generalized high or low bone mass states: susceptibility is systemic and genetic, but lesion formation is anatomically restricted and may depend on local cell state, extracellular matrix, inflammation, and biomechanical context [1,2,3,4,5].

The largest recent population biobank analysis of otosclerosis included 3504 cases and 861,198 controls and reported 27 susceptibility loci [6]. At chromosome 13, the authors specifically described an intronic association signal in *COL4A2*, with associated variants also clustered near *COL4A2-AS2*. The original interpretation therefore already implicated a type IV collagen/basement membrane candidate; the present study does not claim first assignment of this locus. Its intended increment is to test whether additional regulatory, topological, and cell-state layers constrain, rather than replace, the original *COL4A2*-centered annotation.

We focused on the chromosome 13 locus because it combined correlated osteoclast eQTL candidates with publicly accessible chromatin and cellular datasets suitable for cross-layer interrogation. This locus was not selected through a preregistered or prospective ranking of all 27 loci, and this study cannot claim that chr13 was the genome-wide strongest candidate. To address the resulting selection concern transparently, we added a simplified post hoc comparison across all 27 lead loci using the same approximate credible set, mature osteoclast ATAC overlap, and osteoclast eQTL summaries. That contextual screen is not equivalent to the deeper chr13 analysis and is used only to show whether the selected locus is unusual under these limited annotations. The present work is entirely a secondary reanalysis: it generated no new human, animal, or molecular data.

*COL4A2-AS2* is an antisense long non-coding RNA near the risk interval; *COL4A2* encodes an extracellular matrix component; and *RAB20* encodes a small GTPase implicated in vesicular and hypoxic cellular responses. The high linkage disequilibrium of the region makes it essential to separate statistical overlap from mechanistic inference.

**Figure 1:**
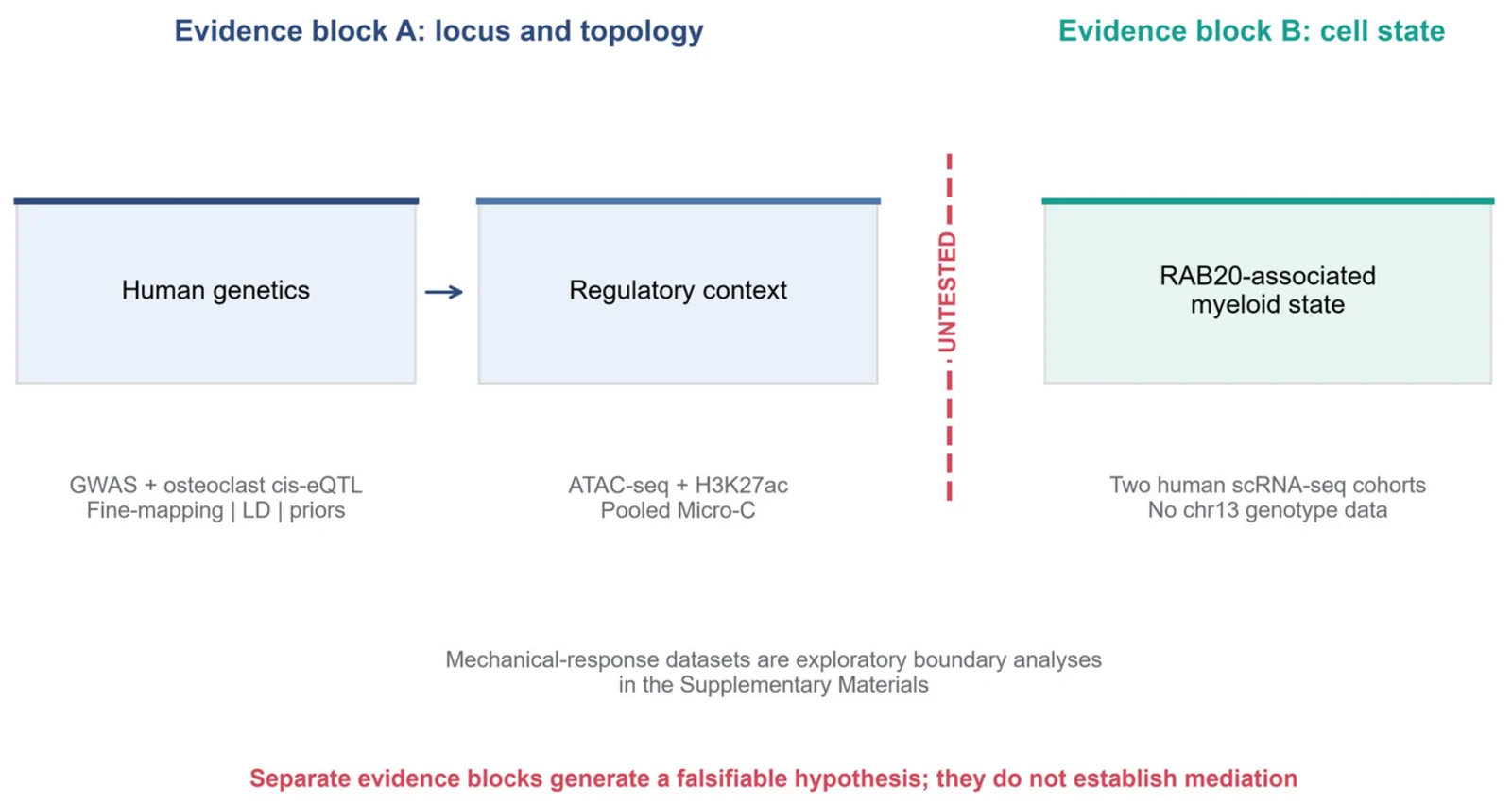
Study design and inferential boundary. The genetic/topological block and the transcriptomic state block are analyzed separately. The dashed divider marks the absence of a dataset that jointly measures chr13 genotype and *RAB20*-associated myeloid state. Mechanical response datasets are reported only as exploratory boundary analyses in the Supplementary Materials.

Our analysis combined fine-mapping, LD and prior sensitivity analysis, gene and tissue controls, osteoclast epigenomic annotation, pooled human Micro-C, and two human single-cell datasets from distinct biological contexts. The genetic/topological and transcriptomic analyses were deliberately kept as non-equivalent evidence blocks. No available dataset jointly measured chr13 genotype and the *RAB20*-associated myeloid state, and no raw dataset sampled an otosclerosis lesion; consequently, their proposed connection is an untested falsifiable hypothesis rather than an observed mediation chain (Figure 1).

## 2. Materials and Methods

### 2.1. Study Design and Evidentiary Framework

We conducted an exploratory secondary analysis of de-identified datasets generated by other investigators. No primary biological or clinical data were collected for this study. No new human participants or animals were recruited. We organized the main text analyses into three evidence levels: (i) human genetic evidence from fine-mapping, colocalization, and negative controls; (ii) regulatory evidence from open chromatin, histone modification, and chromatin contacts; and (iii) cellular state comparison across human single-cell cohorts. Human cyclic strain and mouse whole bone loading datasets formed a separate contextual layer. Evidence from a later level was not used to retrospectively label an upstream association as causal. The datasets differed in assay, donors, and biological context and were not treated as independent replications of one another. Negative and discordant findings were retained as model boundaries. An exploratory evidence-constrained network simulation is reported only in Supplementary Note S2.

### 2.2. Secondary Data Provenance and Source Attribution

All empirical results in this manuscript derive from reanalysis of data generated by other investigators. The otosclerosis summary association statistics originated from the three-biobank GWAS by Rämö et al., comprising FinnGen, the Estonian Biobank, and UK Biobank [6]. Linkage disequilibrium was calculated from European ancestry participants in the 1000 Genomes Project Phase 3 reference panel [7]. Osteoclast cis-eQTL summary statistics were obtained from the GEFOS ‘FastQTL_Osteoclast’ resource generated by Mullin et al. from osteoclast-like cells differentiated in vitro from peripheral blood mononuclear cells of 158 women of European ancestry aged 30–70 years [8].

Regulatory annotations came from NCBI GEO. GSE261280 is the ATAC-seq subseries of a bone mineral density functional genomics study and includes three day-4 RANKL-differentiated human osteoclast donors used here [9]. GSE203354 contains control and RANKL-treated human osteoclast precursor ATAC-seq, and GSE203586 contains H3K27ac ChIP-seq from the same broader RANKL-responsive epigenomic study; both are subseries of GSE203587 and are linked to Bae et al. [10,11,12]. Human osteoclast chromatin loops were obtained from the processed merged Micro-C calls published by Wilson et al. and deposited in Mendeley Data [13,14].

Single-cell discovery used the first breast cancer library from each of 12 participants represented in GSE266330. The published source study profiled 42 bone metastases from eight cancer types; our 12-library subset was defined in this reanalysis to avoid technical duplication and to hold tumor type constant [15]. Cross-context comparison used all four femoral head libraries in GSE169396, obtained from individuals with osteoporosis or osteopenia undergoing hip replacement and originally generated by Qiu et al. [16]. Table 1 distinguishes each source population from its role here. Except for the GWAS, none of these studies were designed to test otosclerosis at chr13.

For disease tissue compatibility, we performed a post hoc audit of the complete thresholded differential gene table published by Ealy et al. for nine otosclerotic and seven non-otosclerotic stapes footplates and of the GPL2986 platform annotation [17]. The original experiment used three technical arrays per biological sample, but no public sample-level expression matrix, repository accession, or supplementary matrix was identified. We therefore retained only the authors’ published fold changes and q-values and calculated no new test statistics. *RAB20*, *CXCL8*, *COL4A2*, *COL4A2-AS2*, *CXCR1*/*IL8RA*, *CXCR2*/*IL8RB*, and *FOSB* were queried. Absence from the 110-gene thresholded table was treated as direction unavailable, not as evidence of no expression or no difference.

**Table 1:** Data layers and inferential roles. Each public dataset is listed with its biological unit, analytical role and principal limitation in this secondary reanalysis. BC, breast cancer; EstBB, Estonian Biobank; EUR, European ancestry; LD, linkage disequilibrium; WGS, whole-genome sequencing.

Layer	Dataset/Resource	Biological Unit	Role in the Manuscript	Principal Limitation
Otosclerosis genetics	Rämö et al. [6]; FinnGen, EstBB, UKBB	3504 cases; 861,198 controls	Defines chr13 risk signal	Summary-level data
LD reference	1000 Genomes Phase 3 EUR [7]	European ancestry reference individuals	Summary fine-mapping and stability split	Internal split, not external replication
External pairwise LD	TOP-LD/TOPMed EUR [18]	High-coverage WGS-derived LD estimates	Tests external stability of the 40-variant high-LD block	Sparse pairs within 1 Mb and r^2^ ≥ 0.2; not a full LD matrix
Osteoclast cis-eQTL	GEFOS FastQTL_Osteoclast; Mullin et al. [8]	Osteoclast-like cells from 158 women of European ancestry	Tests a candidate shared regional signal	In vitro cells; high LD; no valid eQTL FINEMAP confirmation
Chromatin accessibility	GSE261280 [9]; GSE203354 [11]	Three day 4 osteoclast donors; 3 control and 3 RANKL precursor samples	Annotates an open regulatory element	Different stages and peak pipelines
H3K27ac	GSE203586 [12]	Two control and two RANKL samples analyzed	Assesses active enhancer annotation	Processed peaks; incomplete support
3D genome	Wilson et al. [13]; Mendeley Data [14]	Merged calls from four healthy participants	Constrains topology	No donor-level loops or direction
Single-cell discovery	GSE266330 [15]	First BC library from each of 12 patients, selected from 42 metastases	Defines a *RAB20*-associated state in a fixed breast cancer subset	Tumor and treatment context
Single-cell cross-context comparison	GSE169396 [16]	Four femoral head samples	Tests donor direction concordance	Small clinically heterogeneous sample

#### Simplified Comparison Across the 27 Reported GWAS Loci

To place the selected chr13 locus in a broader context, we conducted a post hoc deliberately simplified screen of all 27 lead loci reported by Rämö et al. [6]. For each locus, Wakefield approximate Bayes factor fine-mapping used the same W = 0.04 prior variance to derive a 95% credible set; credible variants were intersected with the GSE261280 mature osteoclast IDR peak set; and a ±1-Mb osteoclast cis-eQTL screen recorded the most significant gene and the number of genes with *p* < 5 × 10^−8^. The screen did not include locus-specific colocalization, matched chromatin topology, or harmonized functional coverage, and it was not preregistered. It therefore provides a descriptive context for locus selection rather than a genome-wide ranking or validation analysis.

### 2.3. Otosclerosis Association, Osteoclast cis-eQTL, and Variant Harmonization

Summary association data for the chr13 otosclerosis locus were obtained from the population biobank GWAS reported by Rämö et al. [6]. Human osteoclast cis-eQTL summary statistics were aligned to GWAS by genomic position and effect allele. Strand-ambiguous variants were excluded when allele frequency could not resolve orientation. Effect directions were expressed relative to the reference otosclerosis risk allele. Regional variants with adequate association and LD information were retained for fine-mapping and colocalization.

### 2.4. Fine-Mapping, LD Diagnostics, and Colocalization

GWAS fine-mapping was performed with FINEMAP v1.0 [19]. SuSiE-RSS [20] was used to allow multiple regional signals and to generate credible sets from summary statistics. Because a complete independent ancestry-matched LD correlation matrix was unavailable, the 1000 Genomes European reference [7] was divided into two non-overlapping internal subsamples (half_A and half_B). These were treated as LD sampling stability analyses, not independent population replication. We compared credible set composition using the Jaccard index and inspected model diagnostics and residual association patterns.

As an external LD sensitivity analysis, we downloaded European ancestry SNV LD estimates from TOP-LD, which was constructed from high-coverage TOPMed whole-genome sequencing [18]. The public chromosome 13 file reports variant pairs within 1 Mb with r^2^ ≥ 0.2, D′, and correlation sign. Among the 40 marginal 95% GWAS credible variants, we compared every reported TOPMed pair with the corresponding 1000 Genomes European estimate. Analysis-defined summaries were Pearson concordance between r^2^ estimates, median absolute r^2^ difference, correlation sign concordance, and the rs7995158–rs9559807 pair. Pairs absent from TOP-LD were treated as censored below the reporting/filtering scheme, not assigned r^2^ = 0. Because this sparse pairwise resource is not a complete positive semidefinite correlation matrix, it was not supplied to FINEMAP or SuSiE-RSS.

For SuSiE-RSS quality control, the estimated summary statistic signal fractions were recorded, the LD matrix was checked for negative eigenvalues, a minimal ridge term was added for numerical stability, and credible set purity and convergence were reviewed. The every-eighth-SNP subset retained 551 of 4389 ordered regional variants to provide an evenly spaced, deterministic and computationally tractable numerical diagnostic. It was not a random sample and did not replace full-region inference. The choice was analysis-defined rather than preregistered.

Colocalization was evaluated using SuSiE-informed colocalization [21,22,23]. The primary shared causal variant prior was p12 = 1 × 10^−5^, with intermediate and stringent sensitivity values of 1 × 10^−6^ and 1 × 10^−7^. We report posterior probability for a shared signal (H4) together with the competing distinct signal posterior (H3), prior sensitivity, and credible set overlap. Cross-method SNP concordance was summarized using the maximum product of FINEMAP GWAS PIP and SuSiE eQTL PIP. eQTL FINEMAP results failed analysis-defined calibration checks because the posterior causal count reached the model cap and implied implausible regional heritability; they were excluded from positive evidence. Approximate conditional analyses were used only to determine whether nominal transcript-level findings were carried by the same haplotype.

Negative controls were analysis-defined for this reanalysis but were not preregistered. *COL4A2*, *RAB20*, and other regional osteoclast eQTLs were compared with *COL4A2-AS2*. GTEx v8 skeletal muscle, tibial artery, whole blood, and cultured fibroblast eQTLs were used as non-osteoclast tissue controls [24]. Micro-C queries from the risk anchor to *COL4A2* and *COL4A2-AS1* transcription start site windows were used as topology controls, and pooled PBMC loops assessed whether contacts were evidently osteoclast-restricted. Negative results constrain specificity but do not prove absence.

### 2.5. Osteoclast Regulatory Annotation

The GWAS credible set was intersected with a mature human osteoclast ATAC-seq peak set from day 4 RANKL-differentiated cells (GSE261280) [9]. Enrichment was assessed by comparing the number of credible variants overlapping ATAC peaks with 10,000 random draws of the same number of variants from 4389 analyzed regional variants. An independent human monocyte-derived osteoclast precursor ATAC-seq dataset (GSE203354) [10,11] was used to assess whether accessibility was visible across control and RANKL-treated samples. Coordinates were converted between genome builds with the UCSC chain where required.

For donor-resolved visualization of the mature osteoclast ATAC-seq signal, paired-end reads from SRR28288271 (GSM8139406), SRR28288270 (GSM8139407), and SRR28288269 (GSM8139408) were aligned to GRCh38 with Bowtie2 v2.5.5 using the very sensitive preset. Proper pairs with MAPQ ≥ 30 were retained, and duplicates were removed with SAMtools markdup. The filtered BAMs were indexed, and 60 kb locus extracts were loaded as separate tracks in IGV v2.19.8; Supplementary Figure S5 displays chr13:110,462,000–110,467,000. Each coverage track uses the same 0–200 vertical range, with individual read alignments shown below. The deposited GSE261280 mature osteoclast IDR peak interval and rs9559807 are overlaid as BED-derived annotation tracks. Coverage was not library size normalized, so the figure supports qualitative locus-level inspection rather than quantitative comparisons across donors or inference of a RANKL treatment effect.

H3K27ac peaks from untreated control and RANKL-treated osteoclast precursors (GSE203586) [10,12] were used to assess active enhancer support. Because only processed peak calls were available and replicate provenance was incomplete, peak presence was not interpreted as quantitative differential acetylation. Candidate variants were ranked by GWAS PIP, distance to the mature osteoclast ATAC summit, LD with the GWAS lead variant, and reproducibility of local accessibility.

### 2.6. Human Osteoclast Micro-C Analysis

Processed merged Micro-C loop calls from osteoclasts generated from four participants and from pooled PBMCs were obtained from Wilson et al. [13,14]. We evaluated seven named edge queries in each cell type at exact coordinates and audited sensitivity to expanded anchors. Source loop FDR values originated from the published merged loop pipeline and were not readjusted across our researcher-defined queries; therefore, query minima were treated descriptively. The public release provided merged loop calls but not a donor-resolved raw contact matrix from which a calibrated contact heatmap could be reconstructed. We consequently visualized the reported loop coordinates as genomic arcs and did not infer contact intensity, directionality, or donor consistency from arc geometry.

### 2.7. Single-Cell Analysis in Tumor-Associated Bone Remodeling

*RAB20* was carried into the single-cell analysis because it was an endpoint of the risk region contact identified in the prespecified regional Micro-C query set, not because it ranked highly in the subsequent transcriptomic screen. The AP1/*CTNNB1* module was analysis-defined from canonical immediate-early AP1 components and *CTNNB1* (*CTNNB1*, *FOS*, *JUN*, *JUNB*, *JUND*, *FOSB*, *FOSL1*, and *FOSL2*) to test a specific myeloid state hypothesis; it was not preregistered. To reduce dependence on that targeted choice, the paired *RAB20*-high versus *RAB20*-low pseudobulk analysis was also evaluated transcriptome-wide, with gene-wise limma-voom statistics and CAMERA pathway tests reported together.

GSE266330 [15] was used as a disease-stressed human bone environment. The first library from each of 12 patients with breast cancer bone metastasis was included to avoid technical duplication. Cells were retained at ≥500 total counts, 200–6000 detected genes, and ≤20% mitochondrial transcripts. Myeloid cells were identified using lineage marker scores. Mature osteoclast-like cells were conservatively defined by expression of at least three of *ACP5*, *CTSK*, *CLCN7*, *TCIRG1*, *ATP6V0D2*, *ITGB3*, *CA2*, and *OSCAR*, a within-patient mature osteoclast score in the upper quartile, and a score greater than the monocyte score. *RAB20* and all tested downstream genes were excluded from the gating definition.

Within each patient, the *RAB20* value, target value, log1p total counts, detected feature count, and mitochondrial fraction were separately rank transformed using average ties. *RAB20* and the target were each regressed on the three ranked covariates, and the Pearson correlation between the two residual vectors was reported as the partial Spearman coefficient. Patients with at least 30 cells entered cross-patient inference; donor coefficients were tested against zero with two-sided Wilcoxon signed-rank tests and adjusted within state target families. The nine gate sensitivity settings crossed minimum mature marker counts of 2, 3, or 4 with within-donor mature score quantiles of 0.50, 0.75, or 0.90.

For paired pseudobulk analysis, mature osteoclast-like cells within each of 10 eligible patients were ranked by normalized *RAB20* expression. The upper and lower thirds were aggregated, producing 20 pseudobulk profiles; the middle third was excluded. Differential expression used voom-limma with a patient blocking term [25,26]. Analysis-defined pathways were tested with CAMERA [27]. Module gene lists and their literature or biological rationale are reported in Supplementary Note S6. These definitions were encoded in archived scripts before final manuscript synthesis but were not preregistered; residual analytic flexibility cannot be excluded.

### 2.8. Cross-Context Assessment in Non-Tumor Human Femoral Head Myeloid Cells

GSE169396, comprising four non-tumor human femoral head samples from individuals with osteoporosis or osteopenia undergoing hip replacement [16], was used as a non-tumor cross-context comparison. A marker-defined myeloid compartment and a pre-osteoclast high subset were analyzed. The latter was the donor-specific upper quartile of a *CSF1R*-independent *TNFRSF11A*/*NFATC1*/*DCSTAMP*/*OCSTAMP* module. Within-donor *RAB20*–ligand associations were residualized for library size, detected features, and mitochondrial fraction. Because only four biological samples were available, the cross-context assessment was based on donor direction concordance for residual correlation and *RAB20*-high versus *RAB20*-low contrasts, rather than cell-level *p* values.

### 2.9. Secretory Program Integration

Candidate ligands discovered in GSE266330 were evaluated in GSE169396 with cognate receptor detection. The directional tier rule and four candidate axes are documented in Supplementary Note S6 and Table S6. The rule was defined in the analysis code before final synthesis but was not prospectively preregistered. Transcript-based ligand expression and receptor detection were treated as hypothesis-generating evidence, not direct protein secretion or receptor activation.

### 2.10. Use of AI-Assisted Tools

OpenAI Codex (used August–October 2026; exact version identifier not recorded) supported script development, debugging, language editing, and submission file formatting as an AI-assisted coding and writing environment. It was not assigned authorship. The authors retained responsibility for the research questions, analytical decisions, source verification, interpretation, code review, and final manuscript, and independently checked the reported outputs against the archived result files.

### 2.11. Statistical Reporting

Two-sided tests were used unless a directional gene set hypothesis had been defined in the analysis code. False discovery rates were controlled within stated analysis families. Effect sizes, donor direction concordance, and uncertainty were emphasized over isolated *p* values. Multiple cells were not treated as independent biological replicates. This study was not preregistered; the terms analysis-defined and predefined refer to code-level definitions used before final cross-dataset synthesis, not to a prospective protocol.

## 3. Results

### 3.1. A Simplified 27-Locus Screen Places chr13 in Context but Does Not Establish Uniqueness

Across the 27 reported lead loci, the approximate 95% credible sets ranged from 2 to 136 variants. Credible variants overlapped mature osteoclast ATAC peaks at 16 loci. At rs7995158, seven credible variants overlapped one ATAC peak and one osteoclast eQTL gene met *p* < 5 × 10^−8^ (*COL4A2-AS2*, *p* = 7.57 × 10^−25^). The chr13 locus was not uniquely strongest: the rs791903 locus had eight credible variants across four ATAC peaks and eight significant osteoclast eQTL genes, while several other loci had more peak intervals or significant eQTL genes than rs7995158 (Supplementary Table S10 and Figure S3). Thus, the screen supports the tractability of the selected locus under available annotations but does not establish locus specificity or prospective priority.

### 3.2. LD and Colocalization Diagnostics Are Compatible with a Candidate Shared Haplotype but Not a Unique Causal Transcript

**Figure 2:**
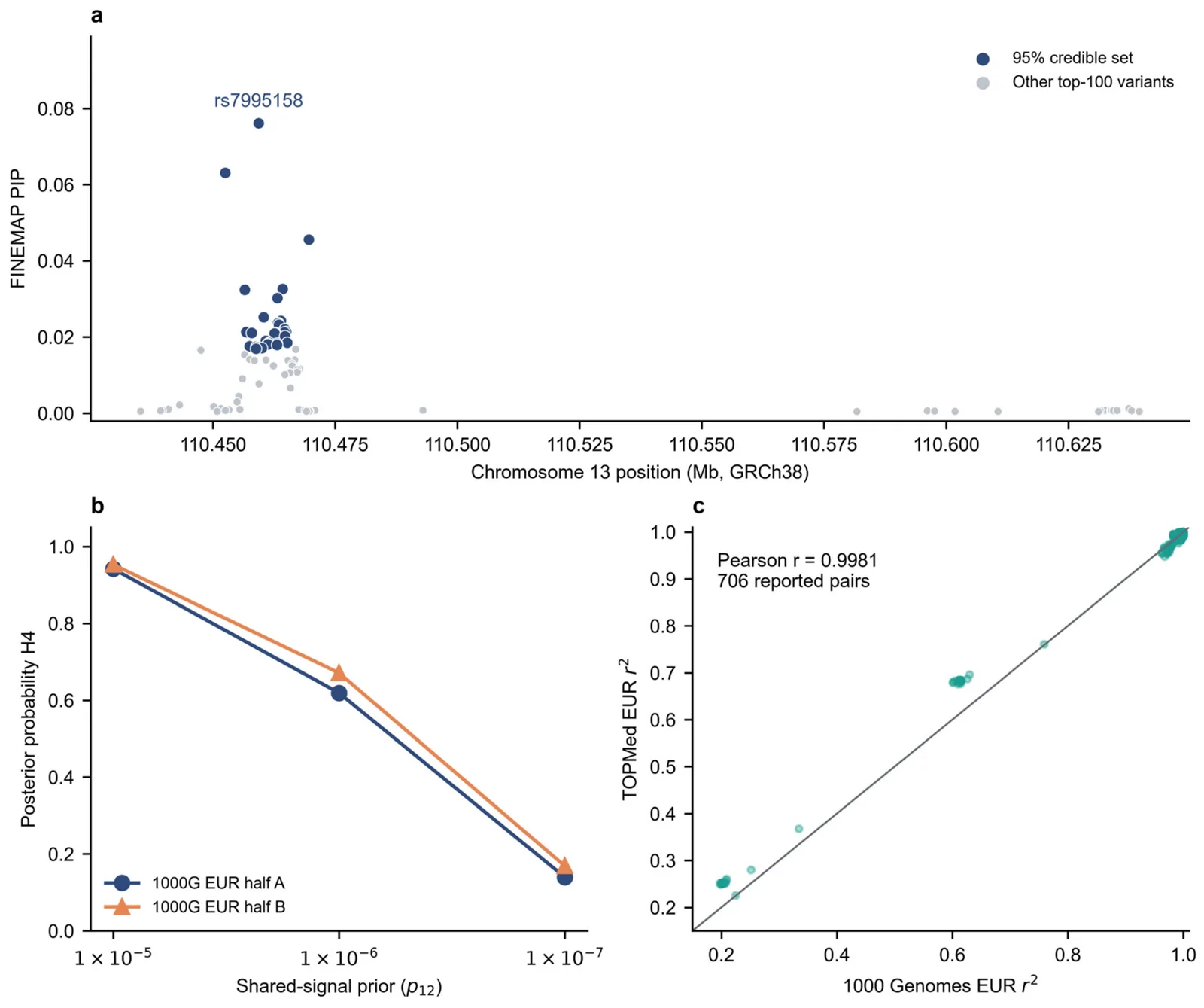
Fine-mapping, prior sensitive colocalization, and external LD concordance. (**a**) FINEMAP posterior inclusion probabilities for the top 100 otosclerosis GWAS variants; dark blue points identify the marginal 95% credible set, and gray points indicate other top-100 variants. (**b**) Colocalization H4 values across shared-signal priors p_12_ = 1 × 10^−5^, 1 × 10^−6^, and 1 × 10^−7^ for the two internally subsampled 1000 Genomes European LD panels (blue circles, half A; orange triangles, half B). (**c**) Comparison of 706 reported TOPMed and 1000 Genomes European r^2^ estimates among the 40 credible variants; the diagonal marks equality. TOPMed supports pairwise LD stability but does not constitute external fine-mapping.

GWAS FINEMAP assigned the highest PIP to rs7995158 (0.0761); the marginal 95% credible set contained 40 variants. SuSiE-informed colocalization showed high H4 under the conventional p12 prior in both internal LD subsamples (half_A, 0.942; half_B, 0.953), but H4 fell to 0.140 and 0.169 at p12 = 1 × 10^−7^. The maximum FINEMAP-GWAS × SuSiE-eQTL PIP products were only 0.00290 and 0.00185. These trajectories show that a shared regional signal is assumption-sensitive and does not robustly identify a single shared causal variant. The eQTL FINEMAP result was excluded after calibration checks indicated an implausible regional model; this exclusion was a safeguard against overinterpretation but removed one potential variant-level triangulation route (Figure 2; Table 2).

SuSiE-RSS numerical diagnostics were acceptable for the main components: the GWAS and eQTL summary statistic signal fraction estimates were 0.0595 and 0.0575, respectively, and main credible set purities exceeded 0.92. The LD audit detected only floating point scale negative eigenvalues (minimum −4.95 × 10^−13^); a ridge of 1.00 × 10^−6^ was sufficient for the diagnostic matrix. These checks reduce concern about gross numerical failure; they do not address ancestry or cohort mismatch. half_A and half_B quantify sampling stability within one reference resource only.

External TOPMed pairwise LD supported the stability of the local haplotype structure. Among the 40 GWAS credible variants, 706 pairs met the TOP-LD reporting criteria and could be compared directly. TOPMed and 1000 Genomes r^2^ estimates had Pearson r = 0.9981, the median absolute r^2^ difference was 0.0030, and all reported pairs had concordant correlation signs. The rs7995158–rs9559807 pair remained strongly linked (TOPMed r^2^ = 0.958; 1000 Genomes r^2^ = 0.971). Thus, the key LD relationship and broader high-LD block were concordant across the two resources. This analysis did not rerun fine-mapping or colocalization because TOP-LD omits pairs with r^2^ < 0.2 and is not a complete LD matrix.

Approximate conditional analyses indicated that most unconditioned support for *COL4A2-AS2*, *COL4A2*, and *RAB20* was carried by the same primary haplotype rather than three separable signals. The risk allele was associated with higher expression of all three transcripts (median harmonized cis-eQTL effects: *COL4A2-AS2*, +0.877; *COL4A2*, +0.463; *RAB20*, +0.427). Because these directions arise from the same haplotype, they are linked observations rather than independent validation. *COL4A2-AS2* was retained as one working genetic entry candidate because the accessible risk element lies near its promoter and its osteoclast eQTL was strongest; the available data did not resolve transcript-specific mediation.

### 3.3. Gene, Tissue, and Topology Controls Constrain Specificity Claims

The osteoclast comparison did not resolve one transcript: at p12 = 1 × 10^−5^, approximate H4 values were 0.946 for *COL4A2-AS2*, 0.931 for *COL4A2*, and 0.850 for *RAB20*, and all decreased under stricter priors. In GTEx v8 controls [24], *COL4A2-AS2* H4 was 0.0818 in skeletal muscle and 0.0223 in tibial artery; corresponding p12 = 1 × 10^−6^ values were 0.00883 and 0.00227. Whole blood *COL4A2* H4 was 0.0132. Cultured fibroblasts had a strong *COL4A2* eQTL but favored distinct causal variants (H3 > 0.999; H4 = 3.31 × 10^−12^). These unequal coverage controls provide context, not proof of osteoclast specificity.

Topological controls were concordant but not definitive. No analysis-defined risk anchor contact was detected with the *COL4A2* or *COL4A2-AS1* transcription start site query. A broader risk-to-*RAB20* loop was present in pooled peripheral blood mononuclear cells, whereas the narrower rs7995158-to-*RAB20*-TSS loop was not. This difference is compatible with, but does not establish, enrichment of the narrower contact in differentiated osteoclasts; the available merged calls cannot support formal cell-type specificity.

### 3.4. Credible Variants Concentrate in One Osteoclast Open Regulatory Element

Seven of 40 credible variants overlapped a single mature osteoclast ATAC peak at chr13:110,463,842–110,464,892 (GRCh38), compared with 145 of 4389 regional variants. The empirical enrichment *p* value was 1.0 × 10^−4^. Because all seven variants were in high LD and occupied one peak, the biological evidence unit was one regulatory element rather than seven independent observations.

rs9559807 was the highest ranked functional candidate within the element: it had the largest PIP among the seven peak-overlapping variants (0.0326), lay 65 bp from the ATAC summit, and was strongly correlated with rs7995158 (r^2^ = 0.971). Independent precursor ATAC-seq documented recurrent but incomplete accessibility around rs9559807 in one of three controls and two of three RANKL samples, while the wider region was accessible in all three samples per condition. These peak calls did not establish RANKL induction.

At the candidate rs9559807 element, coverage and individual alignments were visible for all three day 4 RANKL-differentiated donors, and rs9559807 lies within the deposited IDR peak interval (Supplementary Figure S5). The three coverage tracks share a 0–200 display range. Because raw coverage was not normalized for library size, visual differences between donors are descriptive only. The figure supports inspection of locus signal in each sample, but it is not a statistical assessment of replicate consistency or RANKL induction.

H3K27ac evidence was incomplete. The wider risk peak was covered in one of two controls and one of two RANKL samples; rs9559807 itself was not directly covered, although one RANKL peak ended 39 bp away. We therefore classified the site as an osteoclast open candidate regulatory element, not as a reproducibly active strong enhancer.

### 3.5. Pooled Osteoclast Micro-C Places the Risk/COL4A2-AS2 Interval and RAB20 in a Shared Topological Neighborhood

Across the seven named edge queries and sensitivity grid, merged osteoclast calls contained a contact between the risk ATAC/*COL4A2-AS2* near promoter region and *RAB20* (minimum source loop FDR = 6.1 × 10^−5^) and a contact between the rs7995158 interval and *RAB20*. Because these are pooled, merged loop calls and query minima were not readjusted across our anchors, they were treated as hypothesis-relevant topological observations rather than calibrated evidence for an osteoclast-specific regulatory interaction.

**Figure 3:**
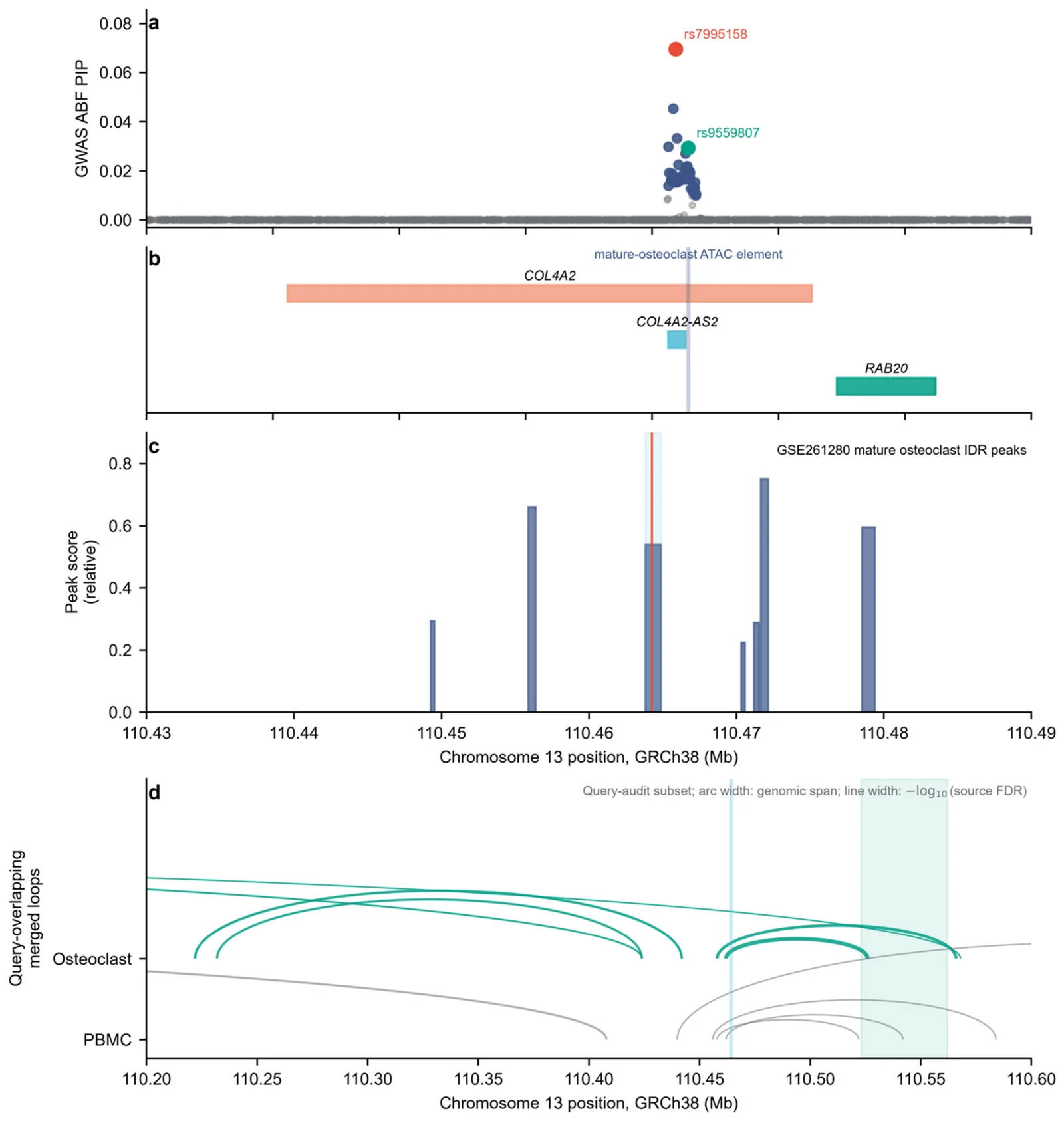
Data proximal view of the chr13 association, regulatory element, and pooled chromatin topology. (**a**) GWAS approximate Bayes factor posterior inclusion probabilities: blue points indicate the 95% credible set, gray points other regional variants, red rs7995158, and green rs9559807. (**b**) Gene intervals from the original analysis annotation: *COL4A2* (orange), *COL4A2-AS2* (cyan), and *RAB20* (green); blue shading marks the mature osteoclast ATAC element. These intervals are gene spans, not exon models. (**c**) GSE261280 mature osteoclast IDR peak calls (blue); cyan shading marks the candidate element and the red line marks rs9559807. Bar heights are relative within the displayed peak file, not quantitative cross-sample accessibility estimates. (**d**) Feature-overlapping public merged Micro-C loop calls for osteoclasts (green) and PBMCs (gray), with cyan shading for the ATAC element and pale green shading for *RAB20*. This query-audit subset is not a complete regional contact map. Arc width represents genomic span and line width reflects the source loop FDR, not contact intensity; arcs may extend beyond the displayed window. The public release did not provide a donor-resolved matrix suitable for a calibrated heatmap.

Related risk region contacts were present in pooled PBMC data, although the exact partners differed. The merged calls therefore identify a topologically active regional neighborhood, not an osteoclast-specific loop. Donor consistency, contact strength, directionality, and allele dependence cannot be inferred from the public merged loop files (Figure 3).

### 3.6. RAB20 Marks an AP1/CTNNB1-Associated Myeloid State in a Human Pathological Bone Environment

The source study profiled 42 bone metastases from eight cancer types [15]. In the present fixed breast cancer subset, 39,633 cells from 12 libraries were considered; 35,892 passed QC, 12,551 were classified as myeloid, and 1116 met the conservative mature osteoclast-like definition. Ten patients had at least 30 osteoclast-like cells. The gate captured a remodeling phenotype: the osteoclast remodeling module was increased relative to monocyte-like cells in all paired samples (median difference +0.314; FDR = 0.00884).

Across all myeloid cells, the partial correlation between *RAB20* and the AP1/*CTNNB1* module was positive in 12/12 patients (median ρ = 0.120; FDR = 0.00816). The result was similar in monocyte-like cells (12/12 positive; median ρ = 0.122; FDR = 0.00632). In the conservative mature osteoclast-like gate, 9/10 patients had positive correlations, but the median effect was smaller and did not survive multiple testing (median ρ = 0.080; nominal *p* = 0.0528; FDR = 0.264). All nine analysis-defined gating schemes yielded a positive median association, while significance diminished as stricter gates reduced cell and donor counts. The association therefore appeared to be a broader myeloid state feature retained as a trend in mature osteoclast-like cells rather than an osteoclast-specific relationship.

**Figure 4:**
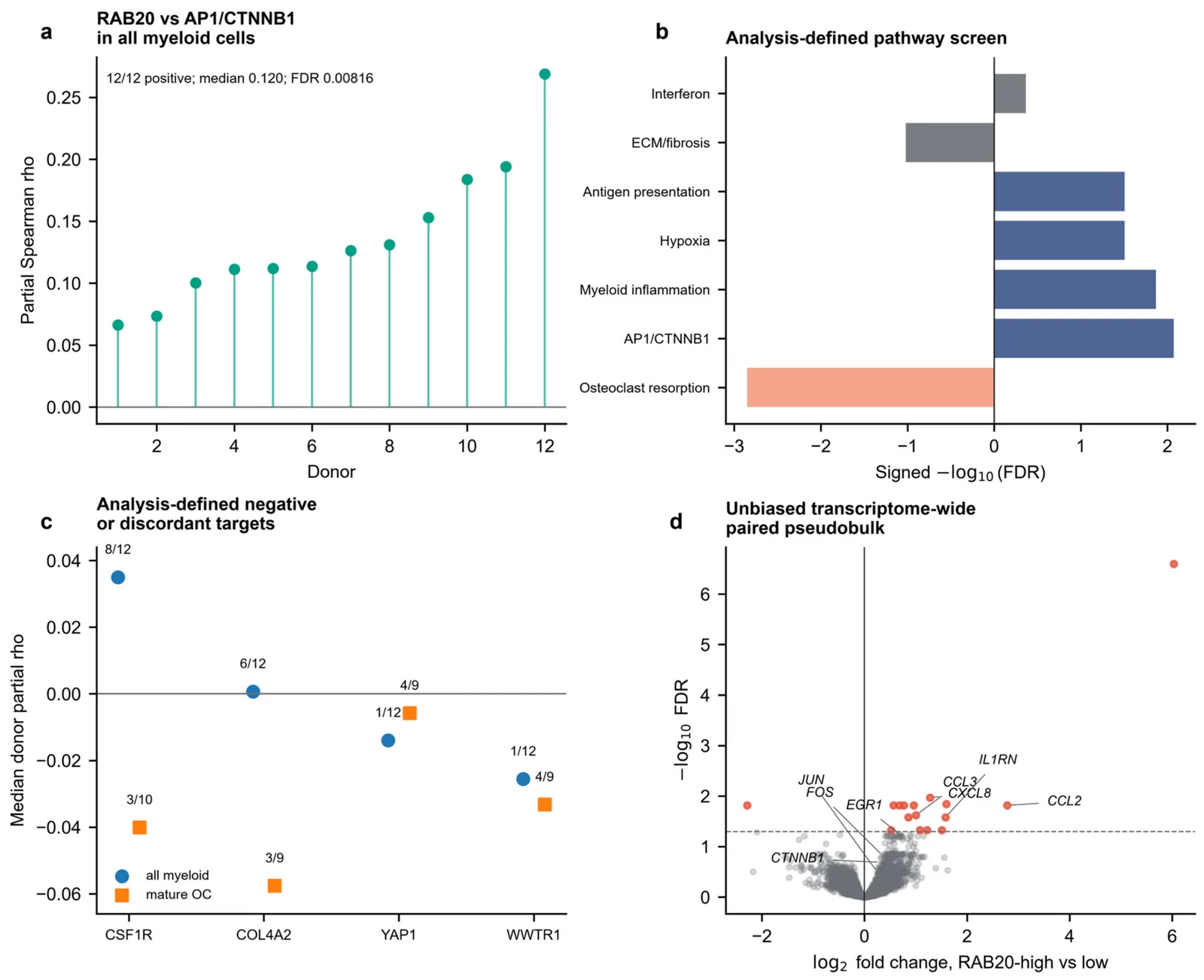
Targeted and transcriptome-wide views of the *RAB20*-associated myeloid state in the breast cancer bone metastasis subset. (**a**) Donor-level partial correlations between *RAB20* and the analysis-defined AP1/*CTNNB1* module in all myeloid cells (12 donors). (**b**) Signed CAMERA results for all tested pathways in the paired pseudobulk analysis (10 eligible donors): blue, FDR < 0.05 and upregulated; orange, FDR < 0.05 and downregulated; gray, FDR ≥ 0.05. (**c**) Median donor partial correlations and direction counts for *CSF1R*, *COL4A2*, *YAP1*, and *WWTR1* in all-myeloid and mature osteoclast-like gates; denominators vary by gene and state and are printed in the panel (blue circles, all myeloid; orange squares, mature osteoclast-like). (**d**) Unbiased paired pseudobulk transcriptome-wide contrast of *RAB20*-high versus *RAB20*-low mature osteoclast-like cells (10 eligible donors). Red points indicate FDR < 0.05 and gray points FDR ≥ 0.05; the dashed line marks FDR = 0.05. These associations are state markers, not causal *RAB20* effects.

The paired pseudobulk contrast provided a more specific view of this state. *RAB20*-high osteoclast-like cells showed increased AP1/*CTNNB1* (CAMERA FDR = 0.00844), myeloid inflammation (FDR = 0.0136), hypoxia, and antigen presentation programs (both FDR = 0.0311), whereas the osteoclast resorption module was lower (FDR = 0.00141). The complementary unbiased gene-wise analysis recovered inflammatory and immediate-early transcripts among the *RAB20*-high changes, while *CSF1R*, *COL4A2*, *YAP1*, and *WWTR1* were negative or discordant across all-myeloid and mature osteoclast-like gates (Figure 4). These results define an association with a broader myeloid state; they do not demonstrate that *RAB20* causes the state or that the state is present in otosclerosis.

### 3.7. CXCL8-CXCR2 Shows the Most Consistent Cross-Context Directional Pattern

*RAB20*-high osteoclast-like cells in GSE266330 expressed higher *CXCL8* (log2 fold change +1.2785; FDR = 0.0107), *CCL2* (+2.7815; FDR = 0.0152), *CCL3* (+1.004; FDR = 0.0240), and *IL1RN* (+1.578; FDR = 0.0264). These findings were assessed for directional concordance in the four non-tumor femoral head samples from donors with osteoporosis or osteopenia.

*CXCL8* showed the clearest cross-context directional concordance. In all-myeloid cells, residual *RAB20*–*CXCL8* correlations were positive in three of four donors and the *RAB20*-high versus low difference was positive in four of four. In the pre-osteoclast high gate, the corresponding direction counts were three of four and four of four, with a median high/low difference of +0.760. *CXCR2* was detected in all four donors, although sparsely. *IL1RN* showed its clearest directional concordance in the pre-osteoclast high state (three of four donors positive for both correlation and high/low difference), consistent with counter regulatory feedback. *CCL3* retained positive residual correlations but lacked a stable high/low contrast. *CCL2* did not show consistent directional concordance and was removed from the central model.

**Figure 5:**
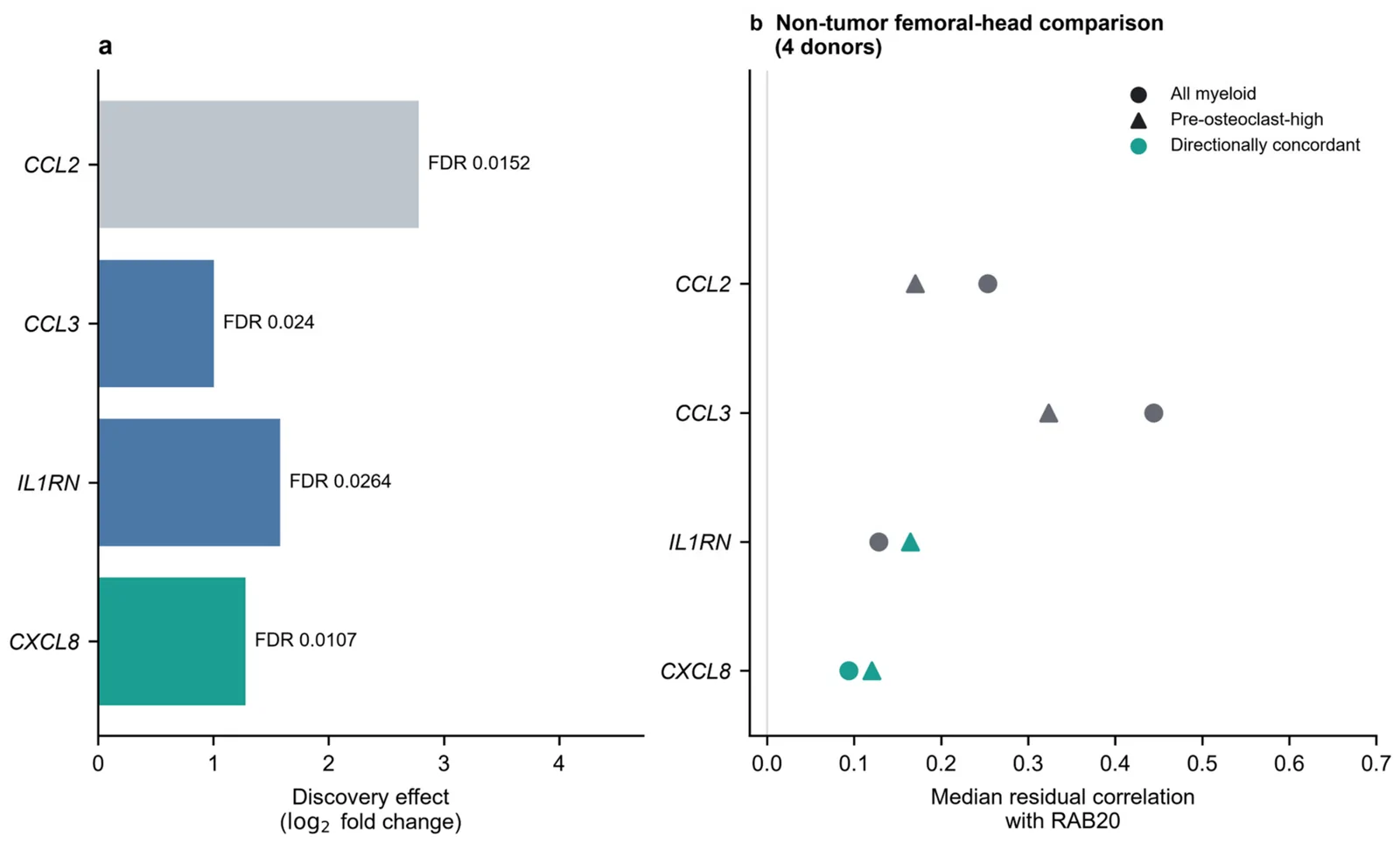
Cross-context comparison of candidate secretory axes. (**a**) Discovery log2 fold changes and FDR values for CXCL8, *IL1RN*, *CCL3*, and *CCL2* in the paired pseudobulk contrast of *RAB20*-high versus *RAB20*-low mature osteoclast-like cells from the tumor-bone cohort; bar colors distinguish the displayed candidates and do not encode a statistical threshold. (**b**) Median donor residual correlations with *RAB20* in four non-tumor femoral head samples from donors with osteoporosis or osteopenia. Circles indicate all myeloid cells and triangles the pre-osteoclast-high gate; green marks the analysis-defined directionally concordant tier and gray other gene–gate combinations. This comparison evaluates direction across biological contexts and is not an independent otosclerosis replication or evidence of protein secretion.

The cross-cohort comparison did not support a broad chemokine program (Supplementary Table S6). It prioritized a falsifiable *RAB20*-associated *CXCL8*-*CXCR2* candidate signaling axis with an *IL1RN* counter regulatory branch. Transcript-based ligand expression and receptor detection evidence cannot establish secretion or receptor activation (Figure 5).

### 3.8. Post Hoc Audit of the Published Otosclerotic Stapes Table

The GPL2986 annotation contained probes mapped to *RAB20*, *CXCL8*, *COL4A2*, *CXCR1*, *CXCR2*, and *FOSB*, but no probe mapped to *COL4A2-AS2*. *RAB20*, *CXCL8*, and *COL4A2* were not listed in the published 110-gene thresholded differential table; without the sample-level matrix, their otosclerosis versus control directions cannot be estimated. The published table reported upregulation of *IL8RA*/*CXCR1* (fold change 2.457; q = 0.0009), *IL8RB*/*CXCR2* (fold change 7.904; q = 0.0009), and *FOSB* (fold change 7.941; q = 0.0178). These thresholded literature-level observations are compatible with the receptor and AP1 components of the proposed state, but they do not validate *RAB20*, *CXCL8*, *COL4A2*, the chr13 locus, or a genotype-to-state pathway (Supplementary Table S9). Table 3 summarizes the evidence and its inferential boundaries.

## 4. Discussion

The central limitation is structural: no raw dataset reanalyzed here sampled the human otic capsule or an otosclerosis lesion, and no dataset jointly measured chr13 genotype and the *RAB20*-associated myeloid state. The genetic/topological block and the transcriptomic state block therefore remain non-equivalent observations. Their connection is a testable integrated hypothesis that could be falsified, not a mechanism supported by an observed mediation chain. The post hoc Ealy stapes audit adds limited disease tissue compatibility for receptor and AP1 components only; it does not validate *RAB20*, *CXCL8*, *COL4A2*, *COL4A2-AS2*, or the chr13-to-state bridge.

The original GWAS already reported an intronic *COL4A2* association and variants near *COL4A2-AS2* [6]. Our incremental contribution is therefore not nearest-gene reassignment. It is an assumption-aware audit showing that the regional eQTL cannot resolve one transcript, that an osteoclast open element lies near *COL4A2-AS2*, that pooled topology connects this neighborhood with *RAB20*, and that *RAB20* indexes an AP1/*CTNNB1*-associated myeloid state in separate datasets. None of these observations establishes that the risk haplotype causes the state.

Historical microarray analysis of nine otosclerotic and seven non-otosclerotic stapes footplates reported inflammatory and interleukin-related expression differences [17]. Our post hoc audit found published upregulation of *CXCR1*, *CXCR2*, and *FOSB*, which is compatible with receptor-level *CXCL8* signaling and an AP1-associated response. *RAB20*, *CXCL8*, and *COL4A2* were absent from the thresholded differential table, and their directions cannot be recovered without the sample-level matrix. This literature-level compatibility check does not provide genotype resolution, cellular localization, mediation, or independent reanalysis of diseased stapes.

*COL4A2* remains a plausible parallel extracellular matrix candidate because the original GWAS signal is intronic, its expression follows the risk haplotype, and type IV collagen contributes to basement membrane organization. The absence of a queried risk anchor–*COL4A2*-TSS loop does not exclude *COL4A2* regulation. Likewise, the *RAB20* state findings do not establish a *RAB20*-to-*COL4A2* fibrotic cascade.

The *RAB20*-associated state also requires careful interpretation. Its association with AP1/*CTNNB1* was strongest across myeloid and monocyte-like cells and weaker within a strict mature osteoclast-like gate. *RAB20*-high cells showed inflammatory, hypoxic, and antigen presentation programs rather than a uniformly stronger terminal resorption program. *RAB20* may therefore index a transition or stress response state that alters communication within the remodeling niche rather than simply increasing osteoclast number or resorptive maturity. Directional agreement for *CXCL8* in non-tumor femoral head myeloid and pre-osteoclast high compartments provides a rationale for examination beyond tumor bone, but the small donor number makes this a prioritization result, not independent disease validation.

Exploratory human cyclic strain and mouse whole bone loading analyses are reported in Supplementary Notes S3 and S5 [28,29,30]. They did not test a powered genotype-by-mechanical-stimulus interaction and did not support direct activation of a *RAB20*-*CXCR2* program. They are retained only as boundary evidence and do not contribute positive support to the central model.

Several design features constrain interpretation: H3/H4 competition was reported across three priors; internal LD subsampling was separated from external pairwise LD sensitivity; the miscalibrated eQTL FINEMAP result was excluded; transcript competition remained unresolved; and PBMC topology was retained as a specificity control. The osteoclast and GTEx comparisons have unequal coverage and provide context rather than proof of osteoclast specificity. The single-cell evidence derives from 12 tumor bone donors and four non-tumor osteoporosis/osteopenia femoral head donors and is consistent with components of a myeloid state hypothesis, not independent otosclerosis validation.

This study has substantial limitations. Colocalization is strongly prior-sensitive, SNP-level concordance is low, and no complete external ancestry-matched LD matrix was available. The 1000 Genomes split evaluates LD sampling stability rather than replication, and TOPMed/TOP-LD provides external pairwise LD stability rather than external fine-mapping. The chr13 locus was selected for data-layer availability without a preregistered all-locus ranking; the added 27-locus screen is post hoc and confirms that chr13 is not uniquely strongest across the limited annotations examined. Micro-C calls are pooled, related PBMC contacts weaken cell-type-specific interpretation, and the public files do not support a donor-resolved heatmap. *COL4A2-AS2* is sparsely captured by scRNA-seq, and transcript-based ligand/receptor analyses do not measure extracellular protein activity.

The essential next step is an allele-aware perturbation series in human monocyte-derived pre-osteoclasts and mature osteoclasts. CRISPR interference of the rs9559807-containing element should be compared directly with targeted *COL4A2-AS2* suppression, with *RAB20*, AP1/*CTNNB1*, *CXCL8*, *IL1RN*, osteoclast differentiation, and matrix remodeling readouts measured in the same donor-genotyped cells. Capture-C or Micro-C in those same cells should test whether the candidate element-*RAB20* contact is allele- or state-dependent. Failure of element perturbation to alter the proposed downstream readouts, or dissociation between *COL4A2-AS2* and *RAB20* effects, would falsify the proposed bridge.

## 5. Conclusions

**Figure 6:**
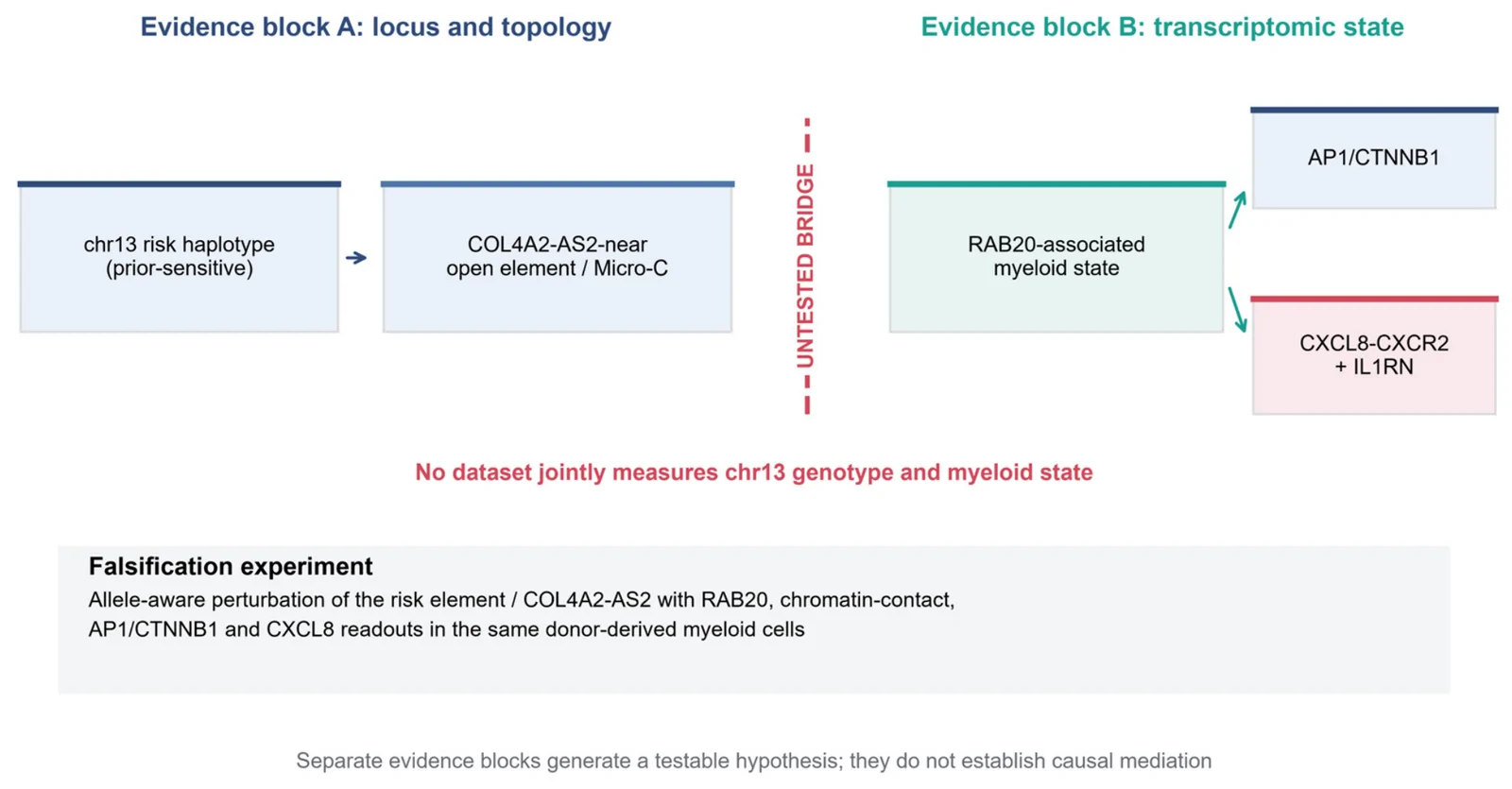
Separate evidence blocks and the untested genotype-to-state bridge. Block A contains prior-sensitive genetic, open chromatin, and pooled topology observations. Block B contains *RAB20*-associated transcriptomic state observations from datasets without chr13 genotypes. The vertical dashed divider marks the absence of any dataset jointly measuring genotype and state. The boxed experiment specifies the joint allele-aware perturbation needed to test or falsify the proposed connection. This is a single schematic; arrows within each evidence block organize observations and do not establish causal direction.

This evidence-calibrated secondary analysis does not identify a causal transcript or establish a genotype-to-cell-state pathway. It defines two separate observations at a selected chr13 otosclerosis locus: a prior-sensitive *COL4A2*/*COL4A2-AS2* regional signal within a topologically active neighborhood, and a *RAB20*-associated AP1/*CTNNB1* myeloid state observed in non-otosclerosis bone datasets. Their proposed connection is explicitly untested. The principal output is a falsifiable experimental design requiring allele-aware perturbation and state readouts in the same human myeloid or osteoclast lineage system (Figure 6).

## Figures and Tables

**Table 2:** Fine-mapping, LD, and colocalization diagnostics. half_A and half_B are non-overlapping internal subsets of the 1000 Genomes European reference and are not independent population replications. CLPP, colocalization posterior probability; eQTL, expression quantitative trait locus; LD, linkage disequilibrium; PIP, posterior inclusion probability.

Diagnostic	half_A	half_B	Interpretation
Reference individuals	252	251	Non-overlapping internal subsets of 1000G EUR; not external replication
H4, p12 = 1 × 10^−5^	0.942	0.953	High under the conventional prior
H4, p12 = 1 × 10^−7^	0.140	0.169	Shared signal support is strongly prior-sensitive
GWAS/eQTL credible set Jaccard index	0.582	0.686	Moderate overlap stable to internal LD sampling
Maximum FINEMAP-GWAS × SuSiE-eQTL CLPP	0.00290	0.00185	Weak individual variant concordance
rs7995158 in both main credible sets	Yes	Yes	Lead region membership stable to internal sampling
Main credible set purity	>0.92	>0.92	Acceptable main component LD coherence
TOPMed/1000G r^2^ concordance	706 reported pairs	r = 0.9981; median absolute difference 0.0030	External pairwise LD stability; not external fine-mapping
rs7995158–rs9559807 r^2^	TOPMed 0.958	1000G 0.971	Key functional candidate relationship is stable

**Table 3:** Evidence summary and inferential boundaries.

Evidence Layer	Current Observation	Permitted Interpretation
Genetic/colocalization	High H4 at p12 = 1 × 10^−5^ but marked decline at p12 = 1 × 10^−7^; low SNP-level concordance	Candidate shared regional signal; no unique variant or transcript
Open chromatin	Seven credible variants in one mature osteoclast ATAC element	Functional prioritization of one element; no causal enhancer claim
Chromatin topology	Pooled merged osteoclast loops connect the region with *RAB20*; related PBMC contacts occur	Topologically active neighborhood; not osteoclast-specific, directional, or allele-dependent
Single-cell state	*RAB20* associates with AP1/*CTNNB1* and inflammatory programs in 12 tumor bone donors	*RAB20* is a state marker in that context; no causal effect or otosclerosis validation
Cross-context comparison	Directional *CXCL8*-*CXCR2* pattern in four osteoporosis/osteopenia femoral head donors	Candidate axis for testing; not independent disease validation or protein activity
Genotype-to-state bridge	No dataset jointly measures genotype and state	Untested falsifiable hypothesis

## Data Availability

All empirical data were generated by other investigators and are available from the sources cited in the manuscript. The otosclerosis GWAS summary statistics were obtained from Rämö et al.; osteoclast cis-eQTL statistics from the GEFOS FastQTL_Osteoclast resource; regulatory data from GEO accessions GSE261280, GSE203354, and GSE203586; human osteoclast Micro-C loops from Wilson et al. and Mendeley Data (https://doi.org/10.17632/gxmswb8nbt.1); single-cell data from GSE266330 and GSE169396; human cyclic-strain data from GSE165874; mechanical-loading data from GSE276391; and external European-ancestry pairwise LD from TOP-LD/TOPMed. Derived tables and analysis scripts are supplied in the reproducibility bundle and in the public GitHub repository https://github.com/weijunma03/otosclerosis-chr13-multiomics (accessed on 5 September 2026). The fixed v1.0.0 release is available at https://github.com/weijunma03/otosclerosis-chr13-multiomics/releases/tag/v1.0.0 (accessed on 5 September 2026) and has been independently archived by Software Heritage as swh:1:rev:82e872321d9b8b2a9285c16e3a7b79e5c7910045 (https://archive.softwareheritage.org/swh:1:rev:82e872321d9b8b2a9285c16e3a7b79e5c7910045, accessed on 5 September 2026). Project name: otosclerosis-chr13-multiomics. Project homepage: https://github.com/weijunma03/otosclerosis-chr13-multiomics (accessed on 5 September 2026). Archived version: v1.0.0, independently preserved as Software Heritage identifier swh:1:rev:82e872321d9b8b2a9285c16e3a7b79e5c7910045. Operating systems: Windows and platform-independent R/Python workflows. Programming languages: R, Python, and PowerShell. Archived R session logs record R v4.1.3; exact historical Python and PowerShell versions were not recorded. Available requirements and package-version records are supplied in the repository and reproducibility bundle. License: MIT. Restrictions for non-academic use: none.

## Supplementary Materials

The following supporting information can be downloaded at: https://www.mdpi.com/article/10.3390/genes17101277/s1, Supplementary Note S1: Expanded genetic diagnostics and negative-control interpretation; Supplementary Note S2: Evidence-constrained virtual perturbation; Note S3: Exploratory donor-paired human cyclic-strain analysis; Note S4: Claims deliberately excluded; Note S5: Mouse whole-bone loading as boundary evidence; Note S6: Statistical definitions, gates, modules and directional tiers; Note S7: Analysis-definition provenance and interpretation scale; Note S8: Published otosclerotic stapes directional audit; Note S9: Simplified context screen across all 27 GWAS loci; Table S1: Colocalization prior sensitivity; Table S2: Non-osteoclast tissue controls; Table S3: External TOPMed pairwise-LD sensitivity; Table S4: GSE165874 donor-level mechanical-response module deltas; Table S5: Evidence grading at the chromosome 13 locus; Table S6. Cross-context assessment of candidate secretory axes; Table S7. Operational evidence categories used for reporting; Table S8. Micro-C query and sensitivity audit; Table S9. Post hoc audit of candidate genes and model-proximal markers in the published Ealy et al. otosclerotic stapes differential table; Table S10. Simplified comparison across all 27 otosclerosis GWAS loci; Figure S1: Evidence-constrained virtual-perturbation sensitivity analysis; Figure S2: Donor-level human cyclic-strain responses; Figure S3. Simplified comparison across the 27 reported GWAS loci; Figure S4. Data-proximal GSE203354 osteoclast-precursor ATAC peak calls around the lifted rs9559807 element in hg19 coordinates; Figure S5. Donor-resolved IGV display of mature-osteoclast ATAC-seq coverage and individual read alignments at GRCh38 chr13:110,462,000-110,467,000 around rs9559807.
