# Supplementary material for: Evidence-Calibrated Reanalysis of an Otosclerosis Locus Generates a Testable Myeloid State Hypothesis
Source: Genes (Basel). 2026 Oct 14;17(10):1277. doi: 10.3390/genes17101277 (PMC13704025; doi:10.3390/genes17101277)
Supplement: Supplementary file 1 [file genes-17-01277-s001.zip › Supplementary_Materials.pdf]

Supplementary Information

Evidence-Calibrated Reanalysis of an Otosclerosis Locus Generates a Testable Myeloid-State Hypothesis

Weijun Ma, Ying Cheng, Heng Li and Hui Lv

Supplementary Note S1. Expanded genetic diagnostics and negative-control interpretation

The two linkage-disequilibrium panels used for FINEMAP, SuSiE-RSS, and colocalization were non-overlapping halves of the same 1000 Genomes European reference and must not be interpreted as ancestry-matched external fine-mapping replication. Across these panels, the conventional shared-signal prior produced H4 values of 0.942 and 0.953, whereas the stringent prior produced H4 values of 0.140 and 0.169. Credible-set Jaccard indices were 0.582 and 0.686, but maximum cross-method SNP-level CLPP remained low (0.00290 and 0.00185). The combined diagnostic pattern supports stability to reference sampling within 1000 Genomes Europeans while showing that the strength and resolution of the shared-signal claim depend materially on model assumptions (Supplementary Table S1).

An external sensitivity analysis used the public TOP-LD European-ancestry chromosome 13 file derived from TOPMed high-coverage whole-genome sequencing. Both the 700.21 MiB LD file and 107.44 MiB annotation file passed their published MD5 checksums. TOP-LD reports pairs within 1 Mb with $r^2 \geq 0.2$, D′, and the sign of the correlation. Among the 40 GWAS credible variants, 706 pairs were available. TOPMed and 1000 Genomes r^2 estimates had Pearson r=0.9981, median absolute difference 0.0030, and 100% sign concordance. For rs7995158–rs9559807, TOPMed r^2 was 0.958 versus 0.971 in 1000 Genomes. These findings externally support the high-LD block but do not provide the complete correlation matrix required to repeat FINEMAP or SuSiE-RSS (Supplementary Table S3).

SuSiE-RSS diagnostic signal-fraction estimates were 0.05949 for the otosclerosis GWAS and 0.05748 for the osteoclast COL4A2-AS2 eQTL. The diagnostic LD matrix contained 551 deterministically thinned variants from the 4,389-variant region; its minimum eigenvalue before regularization was -4.95×10^{-13}, and a ridge term of 1.00×10^{-6} restored numerical positivity. Main credible-set purities exceeded 0.92. Full-regional inference, rather than the thinned diagnostic matrix, supplied the reported locus results. eQTL FINEMAP was excluded because the posterior causal count reached the model cap and the implied regional heritability was implausible.

Transcript competition did not identify a unique mediator. At $p12=1\times10^{-5}$, approximate regional H4 values were 0.946 for COL4A2-AS2, 0.931 for COL4A2, and 0.850 for RAB20. Approximate conditioning substantially reduced the unconditioned signals, consistent with dependence on one primary haplotype. Non-osteoclast controls did not reproduce a strong COL4A2-AS2 result: skeletal-muscle H4 was 0.0818 and tibial-artery H4 was 0.0223 at $p12=1\times10^{-5}$. A strong cultured-fibroblast COL4A2 eQTL favored distinct causal variants (H3>0.999; $H4=3.31\times10^{-12}$), showing that eQTL strength alone is insufficient for locus assignment (Supplementary Table S2).

Supplementary Note S2. Evidence-constrained virtual perturbation

Methods

A compact network was constructed only from directions supported by the preceding analyses: RAB20-high state shifts in AP1/CTNNB1 and hypoxia programs, CXCL8-program induction, CXCR2 feedback, and IL1RN counter-regulation. Unmeasured CXCR2 amplification and IL1RN attenuation gains were each varied over 0.10, 0.25, and 0.40, creating nine analysis-defined combinations. Simulations examined the RAB20-up reference state, RAB20 restoration, CXCR2 feedback blockade, combined restoration and blockade, and removal of IL1RN feedback. Outputs were dimensionless state-propagation scores. They were not causal coefficients, drug-response estimates, or clinical predictions.

Results

Under the central feedback-gain setting of 0.25/0.25, the RAB20-up state produced a modeled net inflammatory score of 0.092. Removing CXCR2 amplification reduced this to 0.011 while leaving upstream AP1/CTNNB1, hypoxia, and ligand programs unchanged. Across all nine gain combinations, CXCR2-feedback

removal changed the output relative to the matched RAB20-up state by −0.130 to −0.032. Removing IL1RN feedback changed it by +0.040 to +0.160. Absolute score signs depended on unmeasured gains, whereas these relative directions were stable.

RAB20 restoration returned all downstream shifts to zero by construction and therefore represents a falsification or rescue scenario rather than evidence for a therapeutic target. These simulations nominate experimental perturbation points only and are deliberately excluded from the main genetic argument (Supplementary Fig. S1).

Supplementary Note S3. Exploratory donor-paired human cyclic-strain analysis

Methods

GSE165874 bulk RNA-seq counts were obtained from GEO. The experiment comprised three 1000 Genomes iPSC donors, paired control and cyclic tensile-strain cultures, and three technical replicates per donor-condition combination. Counts were transformed to log2 counts per million, technical replicates were averaged within donor and condition, and strain-minus-control deltas were calculated. Modules were analysis-defined before examining donor directions: mechanotransduction (PIEZO1, ITGB1, PTK2, RHOA, ROCK1, YAP1, WWTR1, CTNNB1), extracellular matrix (COL4A2, COL1A1, TGFB1, SMAD3), bone remodeling (TNFSF11, TNFRSF11B, NFATC1, CTSK, CLCN7, RUNX2), and chr13 state (COL4A2-AS2, COL4A2, RAB20). Module scores averaged gene-wise standardized expression. Public 1000 Genomes genotypes yielded rs7995158 A-risk-allele dosages for NA18856 and NA19160 only.

Results and interpretation boundary

Mechanotransduction and bone-remodeling module scores increased in all three donors, whereas the chr13-state score decreased in all three. COL4A2-AS2 and RAB20 decreased in every donor; COL4A2 was heterogeneous. The two genotype-matched donors had different rs7995158 risk-allele dosages, but two observations cannot estimate an interaction, dose response, uncertainty interval, or response-eQTL. Technical replicates were not treated as independent biological samples. These results support a mechanically responsive skeletal-cell context while leaving the otosclerosis-risk-genotype-by-mechanics bridge untested (Supplementary Table S4 and Supplementary Fig. S2).

Supplementary Note S4. Claims deliberately excluded

The present data do not justify claims that two independent population LD references constituted replicated colocalization; that FINEMAP confirmed a causal osteoclast eQTL; that COL4A2-AS2, COL4A2, and RAB20 are three independent causal signals; that the Micro-C loop is present independently in all four donors; that the chr13 element is a stable strong enhancer; that RAB20 is osteoclast-specific; that mechanical loading directly activates RAB20–CXCR2; or that CXCR2 blockade is an effective treatment for otosclerosis.

Supplementary Note S5. Mouse whole-bone loading as boundary evidence

GSE276391 contained 20 samples from 10 mice, with loaded and contralateral tibiae under vehicle or dexamethasone conditions (five paired animals per environment). Paired models estimated loading and loading × dexamethasone effects; CAMERA and ROAST tested analysis-defined modules. Neither Rab20 nor the Cxcl1/Cxcl5/Cxcr2 functional module was significant by both methods. Rab20 loading effects were +0.225 under vehicle and +0.153 under dexamethasone; the interaction was −0.072 with intervals spanning zero. Ctnnb1 had a nominal interaction of −0.552 (95% CI −0.927 to −0.177; P=0.00689), but focused FDR=0.0689. This glucocorticoid whole-bone model neither tests genotype interaction nor localizes a lineage and contributes no positive evidence to the central model.

Supplementary Note S6. Statistical definitions, gates, modules and directional tiers

Partial Spearman coefficients were calculated by average-tie rank transformation of RAB20, the target, log1p total counts, detected-feature count and mitochondrial fraction. Ranked RAB20 and target values were separately regressed on the three ranked covariates; Pearson correlation of the residuals was reported. Nine mature-state sensitivity gates crossed minimum marker counts of 2, 3 and 4 with within-donor mature-score quantiles of 0.50, 0.75 and 0.90.

The AP1/CTNNB1 module comprised CTNNB1, FOS, JUN, JUNB, JUND, FOSB, FOSL1 and FOSL2. Additional tested modules were YAP/TAZ/TEAD (YAP1, WWTR1, TEAD1-4, CTGF, CYR61, ANKRD1), osteoclast resorption (ACP5, CTSK, CLCN7, TCIRG1, ATP6V0D2, ITGB3, CA2, OSCAR, MMP9), RANKL/OPG (TNFRSF11A, TNFSF11, TNFRSF11B, TRAF6, NFATC1), ECM/fibrosis (COL1A1, COL1A2, COL3A1, COL4A1, COL4A2, SPARC, FN1, FBN1, MMP2, TGFB1, SMAD3), myeloid inflammation (IL1B, TNF, NFKB1, NFKB2, RELA, CXCL8, S100A8, S100A9), interferon (STAT1, STAT2, IRF1, IRF7, ISG15, IFIT1-3, MX1), antigen presentation (HLA-DRA, HLA-DRB1, HLA-DPA1, HLA-DPB1, CD74, CIITA), and hypoxia (HIF1A, VEGFA, SLC2A1, LDHA, BNIP3, CA9). Modules were biologically curated for this reanalysis and were not preregistered.

The secretory-axis tiers were directional filters, not validated evidence scores. Tier 1 required concordant direction in both all-myeloid and pre-osteoclast-high analyses; Tier 2 indicated state-dependent or partial concordance; Tier 3 indicated discovery-only support. These rules were encoded before final narrative synthesis but were not prospectively registered.

Supplementary Note S7. Analysis-definition provenance and interpretation scale

This study was not preregistered. Gene sets, gates and cross-context directional rules were encoded in archived analysis scripts before final manuscript synthesis, but their development occurred within the same project and may reflect analytic flexibility. The evidence categories in Supplementary Table S7 are reporting labels applied after analysis. They are intended to prevent cross-layer overinterpretation and are not a validated causal-evidence scale.

Supplementary Note S8. Published otosclerotic stapes directional audit

Ealy et al. profiled nine otosclerotic and seven non-otosclerotic stapes footplates on GPL2986, with three technical arrays per biological sample. No public sample-level expression matrix, repository accession or supplementary matrix was identified. We therefore audited only the authors' published 110-gene thresholded table and the GPL2986 annotation. Platform coverage does not supply an expression direction. Absence from the selected differential table means that direction is unavailable under the published thresholds, not that a transcript was absent or unchanged. The audit was post hoc and was not used to revise the central model.

Supplementary Note S9. Simplified context screen across all 27 GWAS loci

A post hoc contextual screen was applied to all 27 lead loci reported by Rämö et al. using the same Wakefield approximate-Bayes-factor prior variance (W=0.04), 95% credible-set definition, GSE261280 mature-osteoclast IDR peaks, and ±1-Mb osteoclast cis-eQTL screen. This deliberately simplified comparison did not perform locus-specific colocalization or integrate matched chromatin topology. Sixteen loci had at least one credible variant overlapping a mature-osteoclast ATAC peak. The rs7995158 locus had seven credible variants in one peak and one significant osteoclast eQTL gene, COL4A2-AS2 ($P=7.57\times10^{-25}$), but it was not uniquely strongest: rs791903 had eight credible variants in four peaks and eight significant eQTL genes. The result supports cross-layer tractability, not prospective locus priority or specificity.

Supplementary Table S1. Colocalization prior sensitivity

LD panel	p12	H4	Interpretation
half_A	1×10^{-5}	0.942	Conventional prior
half_A	1×10^{-6}	0.619	Intermediate prior
half_A	1×10^{-7}	0.140	Stringent prior
half_B	1×10^{-5}	0.953	Conventional prior
half_B	1×10^{-6}	0.671	Intermediate prior
half_B	1×10^{-7}	0.169	Stringent prior

Supplementary Table S2. Non-osteoclast tissue controls

Tissue	Gene	p12	H3	H4
Cultured fibroblasts	COL4A2	1×10^{-5}	>0.999	3.31×10^{-12}
Skeletal muscle	COL4A2-AS2	1×10^{-5}	0.253	0.0818
Tibial artery	COL4A2-AS2	1×10^{-5}	0.205	0.0223
Whole blood	COL4A2	1×10^{-5}	0.278	0.0132

Supplementary Table S3. External TOPMed pairwise-LD sensitivity

Metric	Result	Interpretation
GWAS credible variants requested	40	Marginal 95% FINEMAP set
TOPMed pairs observed	706	Pairs satisfying TOP-LD reporting criteria
TOPMed versus 1000G r^2 Pearson correlation	0.9981	Highly concordant high-LD architecture
Median absolute r^2 difference	0.0030	Small typical between-reference difference
Correlation-sign concordance	100%	No sign reversal among reported pairs
rs7995158–rs9559807 TOPMed r^2	0.958	Key pair remains strongly linked
rs7995158–rs9559807 1000G r^2	0.971	Consistent with TOPMed estimate
Scope boundary	Sparse pairwise LD	Not a complete matrix for FINEMAP/SuSiE-RSS

Supplementary Table S4. GSE165874 donor-level mechanical-response module deltas

Values are strain-minus-control changes in mean standardized module score after averaging technical replicates within donor and condition. They are descriptive and are not independent-sample effect estimates.

Donor	rs7995158 A dosage	Mechanotransduction	Bone remodeling	ECM	chr13 state
NA18855	Not recovered	+0.169	+0.161	+0.057	−0.670
NA18856	1	+1.059	+1.112	−0.134	−1.027
NA19160	2	+0.385	+0.123	−0.197	−0.648

Supplementary Figure S1

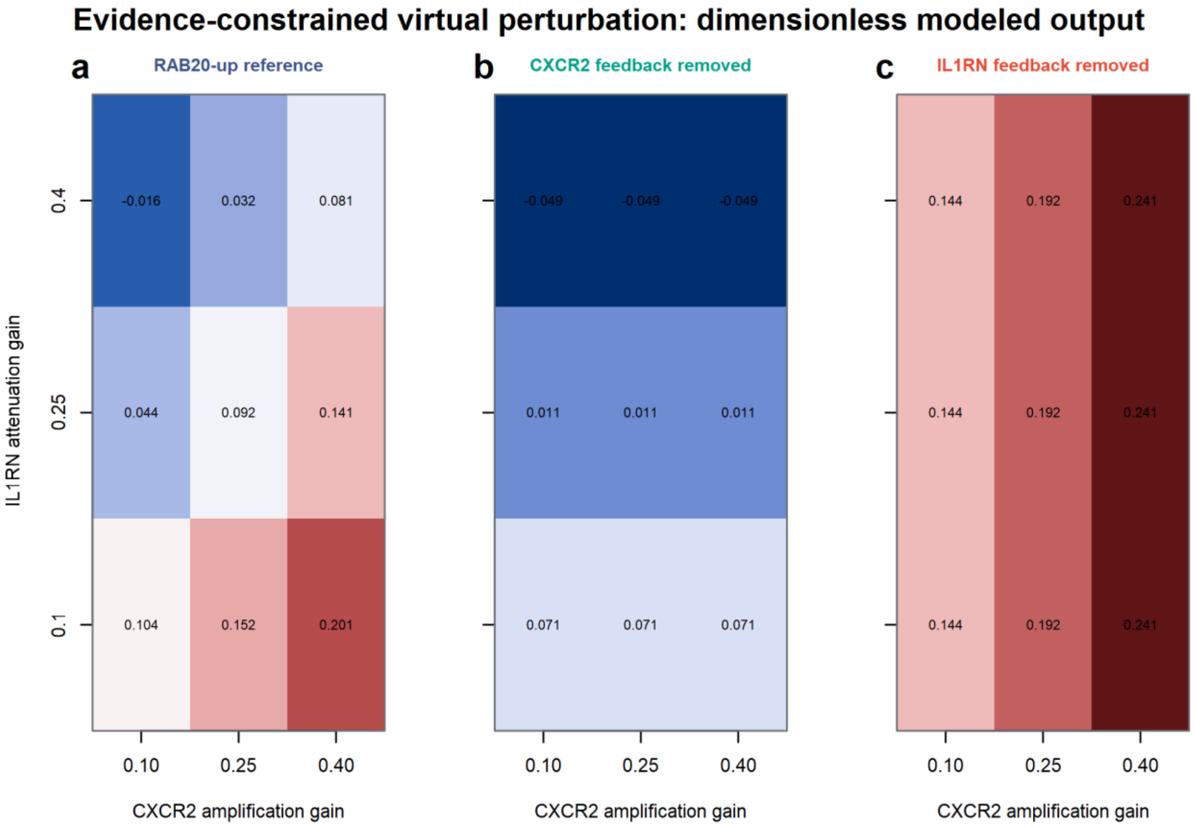

Supplementary Figure S1. Evidence-constrained virtual-perturbation sensitivity analysis. Dimensionless model scores are shown across analysis-defined CXCR2-amplification and IL1RN-attenuation gain combinations. This simulation generates experimental predictions and does not estimate a treatment effect or causal mediation.

Supplementary Figure S2

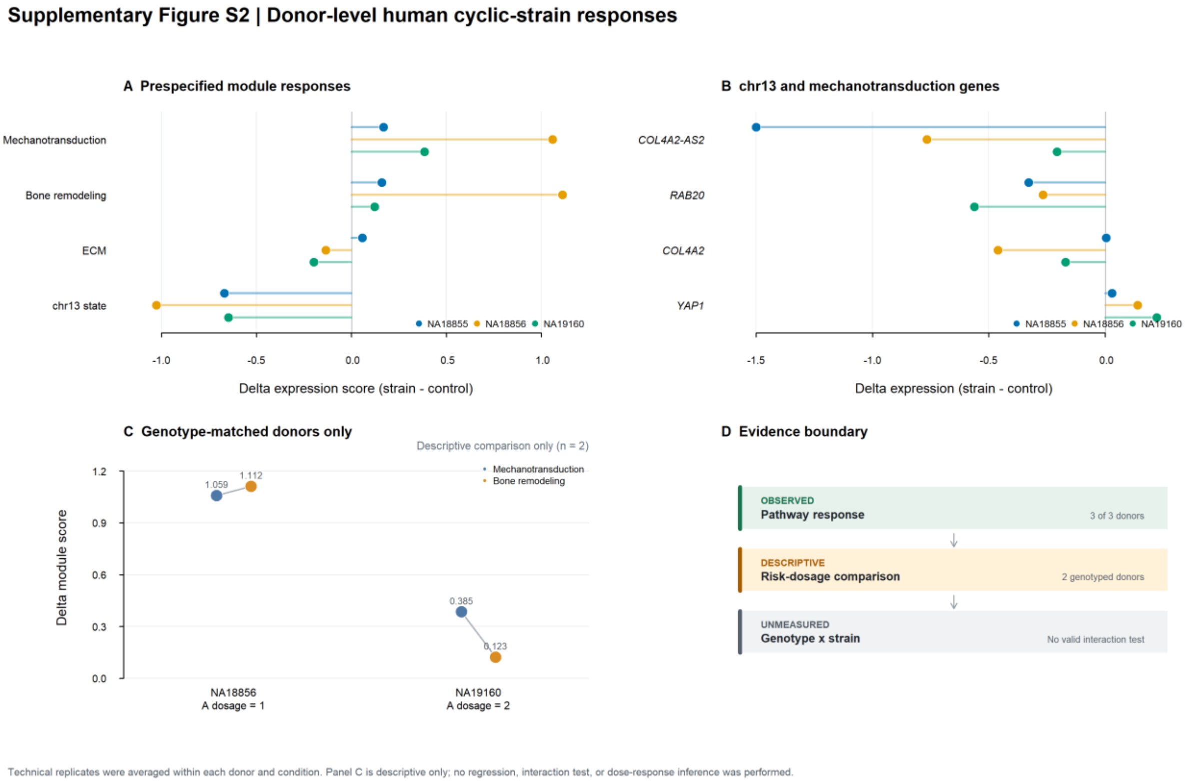

Supplementary Figure S2. Donor-level human cyclic-strain responses. Strain-minus-control changes are shown after technical-replicate averaging within three donors. The two-donor genotype panel is descriptive and does not estimate a genotype-by-strain interaction.

Supplementary Table S5. Evidence grading at the chromosome 13 locus. Evidence layers are kept separate so that genetic association, regulatory annotation, chromatin topology and cellular-state association are not interpreted as a complete causal chain.

Candidate	Genetic evidence	Regulatory/topological evidence	Cellular evidence	Final interpretation
COL4A2-AS2	Strongest osteoclast cis-eQTL among linked transcripts; shared signal is prior-sensitive	Risk element lies near promoter; topology module connects interval to RAB20	Direct single-cell measurement inadequate	Leading genetic-entry candidate, not proven mediator
RAB20	Expression direction follows risk haplotype; not genetically separable	Robust pooled osteoclast contact with risk/near-promoter interval	Cross-context concordant AP1/CTNNB1-associated myeloid state; CXCL8 program	Topology-connected downstream network node
COL4A2	Linked cis-eQTL with concordant risk direction	Credible element lies within gene; no analysis-defined risk-to-TSS Micro-C loop	No stable RAB20-state coupling	Parallel extracellular-matrix candidate
rs9559807 element	PIP=0.0326; r^2=0.971 with rs7995158	65 bp from osteoclast ATAC summit; inconsistent H3K27ac	Not directly tested	Highest-priority functional element/variant candidate

Supplementary Table S6. Cross-context assessment of candidate secretory axes. Tiers reflect directional agreement across the tumor-bone discovery and non-tumor femoral-head cross-context dataset; they do not represent protein secretion, receptor activation or treatment effects.

Axis	Tumor-bone discovery	Non-tumor femoral-head cross-context comparison	Updated interpretation
CXCL8–CXCR2	CXCL8 FDR=0.0107; receptor context present	Directionally concordant in all-myeloid and preOC-high gates; CXCR2 detected in 4/4 donors	Tier 1 directionally concordant candidate axis
IL1RN–IL1R1/IL1RAP	IL1RN FDR=0.0264	Most consistent in preOC-high gate; receptors detected in 4/4	Tier 2 state-dependent counter-regulation
CCL3–CCR1/CCR5	CCL3 FDR=0.0240	Correlation direction consistent, high–low contrast unstable	Tier 2 partial association
CCL2–CCR2	CCL2 FDR=0.0152	Did not meet the directional concordance rule	Tier 3 discovery-specific; excluded from central model

Supplementary Table S7. Operational evidence categories used for reporting. These categories describe the relation between data and claim; they are not a preregistered or validated scoring system.

Category	Operational meaning	Permitted wording
Observed within layer	Directly computed association, contact or annotation with stated inferential unit and multiplicity scope	Observed/associated; no mediation language
Assumption-sensitive support	Direction or regional pattern supported but materially dependent on priors, LD, pooling, coverage or model choice	Compatible with/supports, with limitation stated
Cross-dataset hypothesis	Connection requires linking datasets that do not jointly measure exposure and outcome	Hypothesis-generating/testable; not integrated mediation
Not tested	Required genotype, tissue, perturbation or protein measurement is absent	Explicitly untested

Supplementary Table S8. Micro-C query and sensitivity audit. Source-loop FDR values were generated by the original merged-loop pipeline and were not re-adjusted across these researcher-defined queries.

Query design	Levels	Number	Interpretive scope
Named edges	risk ATAC→RAB20 gene/TSS; rs7995158→RAB20 TSS; AS2 TSS→RAB20 gene/TSS; risk ATAC→COL4A2 TSS; risk ATAC→AS1 TSS	7	Includes two topology negative controls
Cell type	Merged osteoclast; pooled PBMC	2	No donor-level differential-contact test
Symmetric anchor padding	0, 250, 500, 1,000, 2,000, 4,000 bp	6	Defines exact and expanded anchors
Source-loop FDR threshold	0.1, 0.05, 0.01, 0.001	4	Sensitivity thresholds, not new FDR correction
Total grid	7 × 2 × 6 × 4	336	Minimum source FDR is descriptive across queries

Supplementary Table S9. Post hoc audit of candidate genes and model-proximal markers in the published Ealy et al. otosclerotic stapes differential table. Fold changes and q-values are reproduced from the publication; no statistic was recomputed.

Target	GPL2986 coverage	Published table status	Direction/statistic	Interpretation
RAB20	Mapped probe	Not listed among 110 thresholded genes	Unavailable	Does not test the RAB20 tissue direction
CXCL8/IL8	Mapped probe	Not listed among 110 thresholded genes	Unavailable	Does not test ligand direction
COL4A2	Mapped probe	Not listed among 110 thresholded genes	Unavailable	Does not test extracellular-matrix direction
COL4A2-AS2	No mapped GPL2986 probe found	Not assessable	Unavailable	Legacy platform cannot evaluate the antisense RNA
CXCR1/IL8RA	Mapped probe	Reported as significant	Up; fold change 2.457; q=0.0009	Receptor-level compatibility only
CXCR2/IL8RB	Mapped probe	Reported as significant	Up; fold change 7.904; q=0.0009	Receptor-level compatibility only
FOSB	Two mapped probes	Reported as significant	Up; fold change 7.941; q=0.0178	AP1-component compatibility only

Supplementary Table S10. Simplified comparison across all 27 otosclerosis GWAS loci. Raw values are descriptive; no cross-locus significance test or prospective ranking was performed.

Lead SNP	Chr	Lead P	95% CS n	CS variants in OC ATAC	Distinct OC ATAC peaks	Top OC eQTL gene	Top OC eQTL P	OC eQTL genes $P<5\times10^{-8}$
rs201694067	1	4.3e-09	38	3	2	LYPLAL1	2.87e-06	0
rs11683921	2	3.23e-10	17	0	0	RGPD8	2.67e-23	3
rs4917	3	3.35e-12	5	0	0	CRYGS	2.75e-06	0
rs181831514	4	2.87e-12	86	1	1	NUDT9	3.95e-06	0
rs791903	6	1.15e-13	67	8	4	HLA-DMA	3.62e-69	8
rs13192457	6	4.8e-11	136	2	2	CDC5L	2.69e-06	0
rs4464751	6	3.58e-08	62	2	2	SLC17A5	1.06e-10	1
rs39375	7	3.21e-14	44	0	0	SH2B2	7.65e-05	0
rs485107	8	2.2e-09	27	3	2	SGK223	1.38e-15	2

Lead SNP	Chr	Lead P	95% CS n	CS variants in OC ATAC	Distinct OC ATAC peaks	Top OC eQTL gene	Top OC eQTL P	OC eQTL genes $P<5\times10^{-8}$
rs4876361	8	0	3	0	0	MED30	7.39e-05	0
rs4877080	9	3.6e-09	28	1	1	CTSL	8.39e-07	0
rs7024182	9	3.83e-08	29	1	1	INVS	1.7e-07	0
rs947091	10	1.8e-15	11	1	1	DDX10P1	3.75e-12	1
rs116948503	11	0	18	4	2	AP003068.23	2.44e-19	4
rs553652	11	9.24e-10	13	3	3	CCDC82	6.69e-06	0
rs73172296	13	1.18e-09	21	2	2	DNAJC15	2.28e-36	1
rs2762049	13	2.34e-09	26	0	0	SETDB2	1.99e-16	3
rs7995158	13	1.8e-15	49	7	1	COL4A2-AS2	7.57e-25	1
rs1013650	14	0	86	1	1	APOPT1	3.63e-09	2
rs2118612	15	3.34e-08	4	0	0	IQCH-AS1	2.26e-16	2
rs67284550	16	1.43e-10	2	0	0	FAHD1	2.42e-11	3
rs4636903	16	7.32e-11	19	0	0	RP11-212I21.2	1.37e-05	0
rs11867466	17	0	2	0	0	WIPI1	2.55e-07	0
rs66487118	19	1.76e-08	11	0	0	ZNF266	1.25e-14	2
rs8105161	19	2.71e-08	59	6	4	LTBP4	1.28e-09	1
rs6066131	20	4.91e-08	30	0	0	ZNF334	1.12e-23	2
rs6066825	20	1.2e-08	40	3	1	ARFGEF2	5.25e-06	0

Supplementary Figure S3

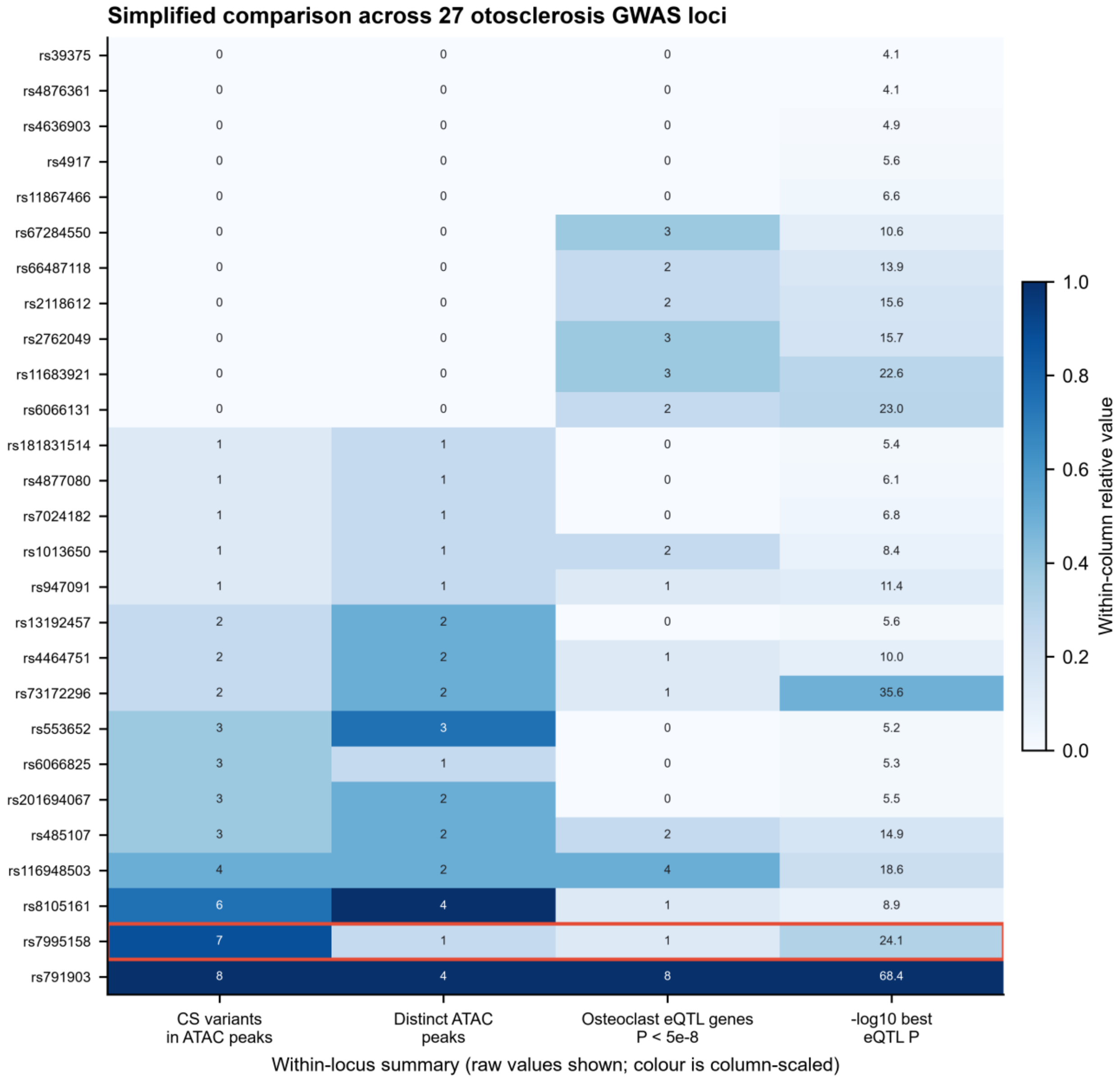

	CS variants in ATAC peaks	Distinct ATAC peaks	Osteoclast eQTL genes P < 5e-8	-log10 best eQTL P
rs39375	0	0	0	4.1
rs4876361	0	0	0	4.1
rs4636903	0	0	0	4.9
rs4917	0	0	0	5.6
rs11867466	0	0	0	6.6
rs67284550	0	0	3	10.6
rs66487118	0	0	2	13.9
rs2118612	0	0	2	15.6
rs2762049	0	0	3	15.7
rs11683921	0	0	3	22.6
rs6066131	0	0	2	23.0
rs181831514	1	1	0	5.4
rs4877080	1	1	0	6.1
rs7024182	1	1	0	6.8
rs1013650	1	1	2	8.4
rs947091	1	1	1	11.4
rs13192457	2	2	0	5.6
rs4464751	2	2	1	10.0
rs73172296	2	2	1	35.6
rs553652	3	3	0	5.2
rs6066825	3	1	0	5.3
rs201694067	3	2	0	5.5
rs485107	3	2	2	14.9
rs116948503	4	2	4	18.6
rs8105161	6	4	1	8.9
rs7995158	7	1	1	24.1
rs791903	8	4	8	68.4

Supplementary Figure S3. Simplified comparison across the 27 reported GWAS loci. Raw values are displayed in cells; color is scaled within each column and is not comparable across columns. The red outline marks rs7995158. This post hoc screen provides selection context and is not a genome-wide multi-omic ranking.

Supplementary Figure S4

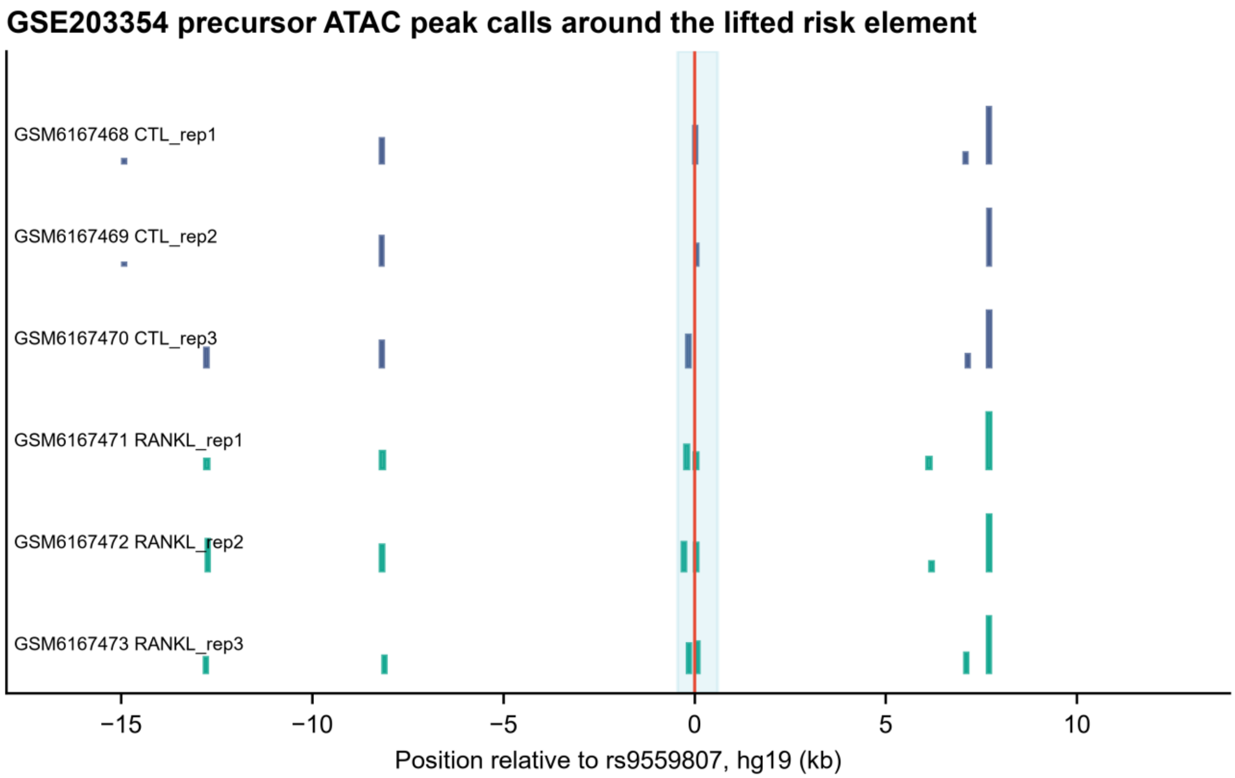

Supplementary Figure S4. Data-proximal GSE203354 osteoclast-precursor ATAC peak calls around the lifted rs9559807 element in hg19 coordinates. Peak heights are scaled within each processed sample and show peak-call geometry, not quantitative accessibility across samples. Control and RANKL-treated samples are shown separately; the panel does not estimate a treatment effect.

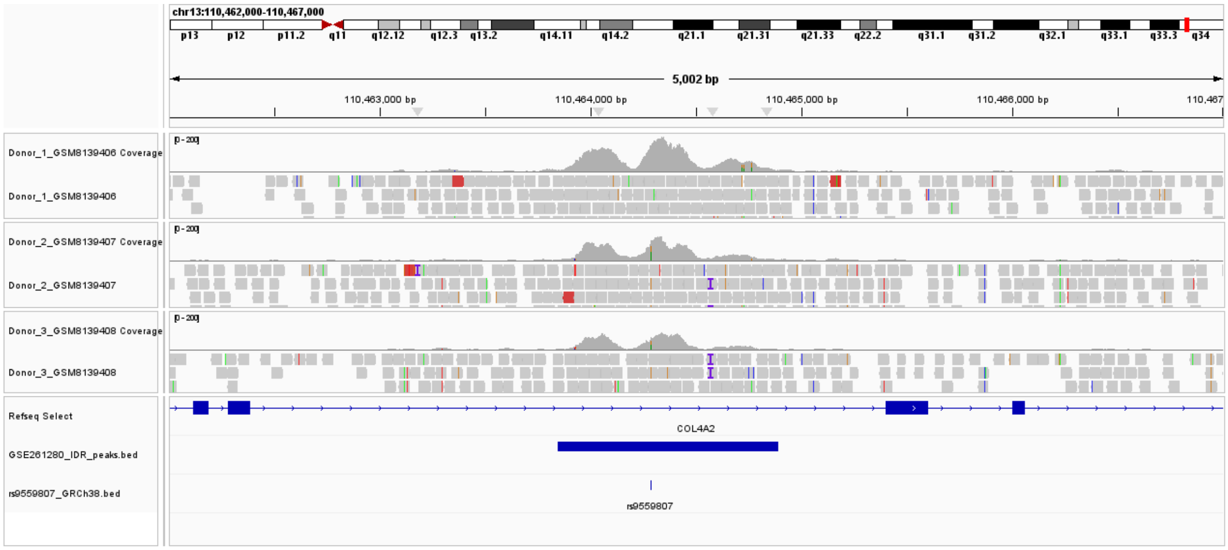

Supplementary Figure S5. Donor-resolved IGV display of mature-osteoclast ATAC-seq coverage and individual read alignments at GRCh38 chr13:110,462,000-110,467,000 around rs9559807. The three day-4 RANKL-differentiated human osteoclast donors from GSE261280 are shown separately (Donor 1/GSM8139406/SRR28288271; Donor 2/GSM8139407/SRR28288270; Donor 3/GSM8139408/SRR28288269). Each coverage track uses a shared 0-200 vertical range. RefSeq Select annotates COL4A2. The GSE261280_IDR_peaks.bed track marks the deposited mature-osteoclast IDR peak at chr13:110,463,842-110,464,892 (BED 0-based, half-open), and rs9559807_GRCh38.bed marks rs9559807 at chr13:110,464,288. These BED tracks are annotations, not signal tracks. The BAMs are regional extracts from donor-specific full-depth filtered GRCh38 alignments. The shared display range is not library-size normalization; coverage is shown for qualitative locus-level inspection and should not be treated as a normalized quantitative comparison across donors.
